# Protein Science Meets Artificial Intelligence: A Systematic Review and a Biochemical Meta-Analysis of an Inter-Field

**DOI:** 10.3389/fbioe.2022.788300

**Published:** 2022-07-07

**Authors:** Jalil Villalobos-Alva, Luis Ochoa-Toledo, Mario Javier Villalobos-Alva, Atocha Aliseda, Fernando Pérez-Escamirosa, Nelly F. Altamirano-Bustamante, Francine Ochoa-Fernández, Ricardo Zamora-Solís, Sebastián Villalobos-Alva, Cristina Revilla-Monsalve, Nicolás Kemper-Valverde, Myriam M. Altamirano-Bustamante

**Affiliations:** ^1^ Unidad de Investigación en Enfermedades Metabólicas, Centro Médico Nacional Siglo XXI, Instituto Mexicano del Seguro Social, Mexico City, Mexico; ^2^ Instituto de Ciencias Aplicadas y Tecnología (ICAT), Universidad Nacional Autónoma de México (UNAM), Mexico City, Mexico; ^3^ Instituto de Investigaciones Filosóficas, Universidad Nacional Autónoma de México (UNAM), Mexico City, Mexico; ^4^ Instituto Nacional de Pediatría, Mexico City, Mexico

**Keywords:** artificial intelligence, proteins, protein design and engineering, machine learning, deep learning, protein prediction, protein classification, drug design

## Abstract

Proteins are some of the most fascinating and challenging molecules in the universe, and they pose a big challenge for artificial intelligence. The implementation of machine learning/AI in protein science gives rise to a world of knowledge adventures in the workhorse of the cell and proteome homeostasis, which are essential for making life possible. This opens up epistemic horizons thanks to a coupling of human tacit–explicit knowledge with machine learning power, the benefits of which are already tangible, such as important advances in protein structure prediction. Moreover, the driving force behind the protein processes of self-organization, adjustment, and fitness requires a space corresponding to gigabytes of life data in its order of magnitude. There are many tasks such as novel protein design, protein folding pathways, and synthetic metabolic routes, as well as protein-aggregation mechanisms, pathogenesis of protein misfolding and disease, and proteostasis networks that are currently unexplored or unrevealed. In this systematic review and biochemical meta-analysis, we aim to contribute to bridging the gap between what we call *binomial* artificial intelligence (AI) and protein science (PS), a growing research enterprise with exciting and promising biotechnological and biomedical applications. We undertake our task by exploring “the state of the art” in AI and machine learning (ML) applications to protein science in the scientific literature to address some critical research questions in this domain, including What kind of tasks are already explored by ML approaches to protein sciences? What are the most common ML algorithms and databases used? What is the situational diagnostic of the AI–PS inter-field? What do ML processing steps have in common? We also formulate novel questions such as Is it possible to discover what the rules of protein evolution are with the binomial AI–PS? How do protein folding pathways evolve? What are the rules that dictate the folds? What are the minimal nuclear protein structures? How do protein aggregates form and why do they exhibit different toxicities? What are the structural properties of amyloid proteins? How can we design an effective proteostasis network to deal with misfolded proteins? We are a cross-functional group of scientists from several academic disciplines, and we have conducted the systematic review using a variant of the PICO and PRISMA approaches. The search was carried out in four databases (PubMed, Bireme, OVID, and EBSCO Web of Science), resulting in 144 research articles. After three rounds of quality screening, 93 articles were finally selected for further analysis. A summary of our findings is as follows: regarding AI applications, there are mainly four types: *1*) genomics, *2*) protein structure and function, *3*) protein design and evolution, and *4*) drug design. In terms of the ML algorithms and databases used, supervised learning was the most common approach (85%). As for the databases used for the ML models, PDB and UniprotKB/Swissprot were the most common ones (21 and 8%, respectively). Moreover, we identified that approximately 63% of the articles organized their results into three steps, which we labeled *pre-process*, *process*, and *post-process*. A few studies combined data from several databases or created their own databases after the pre-process. Our main finding is that, as of today, there are no research road maps serving as guides to address gaps in our knowledge of the AI–PS binomial. All research efforts to collect, integrate multidimensional data features, and then analyze and validate them are, so far, uncoordinated and scattered throughout the scientific literature without a clear epistemic goal or connection between the studies. Therefore, our main contribution to the scientific literature is to offer a road map to help solve problems in drug design, protein structures, design, and function prediction while also presenting the “state of the art” on research in the AI–PS binomial until February 2021. Thus, we pave the way toward future advances in the synthetic redesign of novel proteins and protein networks and artificial metabolic pathways, learning lessons from nature for the welfare of humankind. Many of the novel proteins and metabolic pathways are currently non-existent in nature, nor are they used in the chemical industry or biomedical field.

## Introduction

Protein science witnesses the most exciting and demanding revolution of its own field; the magnitude of its genetic–epigenetic—molecular networks, inhibitors, activators, modulators, and metabolite information—is astronomical. It is organized in an open “protein self-organize, adjustment and fitness space”; for example, a protein of 100 amino acids would contain 20^100^ variants, and a process of searching–finding conformations in a protein of 100 amino acids can adopt ∼10^46^ conformation and a unique native state, the protein data exceeding many petabytes (1 petabyte is 1 million gigabytes) (Kauffman, 1992).

Therefore, the use of artificial intelligence in protein science is creating new avenues for understanding the ways of organizing and classifying life within its organisms to eventually design, control, and improve this organization. In this respect, protein synthesis is a case in point. Indeed, the discovery of the underlying mechanism of protein synthesis is an inter-field discovery, that is, “a significant achievement of 20th century biology that integrated results from two fields: molecular biology and biochemistry” (Baetu, 2015). More recently, the field of *protein science* is, in turn, another inter-field enterprise, this time between molecular biology and computer science, or better said, between a cross-functional team of researchers (biochemists, protein scientists, protein engineers, system biology scientists, bioinformatics, between others). Nowadays, it is possible to classify, share, and use a significant number of structural biology databases helping researchers throughout the world. Once the mechanism of DNA for protein synthesis is deduced, it will then be possible to replicate it *via* computational strategies through artificial intelligence (AI) and machine learning (ML) algorithms that can provide important information such as pattern recognition, nearest neighbors, vector profiles, back propagation, among others. AI has been used to exploit this novel knowledge to predict, design, classify, and evolve known proteins with improved and enhanced properties and applications in protein science (Paladino et al., 2017; Wardah et al., 2019;Cheng et al., 2008; Bernardes and Pedreira, 2013), which, in turn, makes its way to solve complex problems in the “fourth industrial revolution” and open new areas of protein research, growing at a very fast speed.

The techniques of machine learning are a subfield of AI, which has become popular due to the linear and non-linear processed data and the large amount of available combinatorial spaces. As a result, sophisticated algorithms have emerged, promoting the use of neural networks (Gainza et al., 2016) However, in spite of the large amount of research done in protein science, as far as we know, there are neither systematic reviews nor any biochemical meta-analysis in the scientific literature informing, illuminating, and guiding researchers on the best available ML techniques for particular tasks in protein science; albeit there have been recent reviews such as the work of AlQuraishi (2021), Dara et al. (2021), and Hie and Yang (2022), which prove that this inter-field is on evolution. By a biochemical meta-analysis, we mean an analysis resulting from two processes: **identification** and **prediction**. The former consists of identifying AI applications into the protein field where we classify and identify active and allosteric sites, molecular signatures, and molecular scaffolding not yet described in nature.

Each structural signature, pattern, or profile constitutes a singular part of the whole “lego-structure-kit” that is the protein space that includes the catalytic task space and shape space, which Kauffman (1992) defines as an abstract representation or mapping of all shapes and chemical reactions that can be catalyzed onto a space of task. The latter process is an analysis of the resulting predictions of structures, molecular signatures, regulatory sites, and ligand sites. Both processes are related to each other in the sense that the proteins in the identification process are searching targets of the 3D-structure for the prediction process that predicts the protein conformation multiple times from a template family or using model-free approach. The biochemical meta-analysis includes formulating the research question, searching and classifying protein tasks in the selected studies, gathering AI–PS information from the studies, evaluating the quality of the studies, analyzing and classifying the outcomes of studies, building up tables and figures for the interpretation of evidence, and presenting the results.

This study puts forward the use of ML classes and methods to address complex problems in protein science. Our point of departure is the state of the art of the AI–PS binomial; by binomial, we mean a biological name consisting of two terms that are partners in computational science as well as in biomedical or biotechnological science as a “two-feet principle” in order to understand, enhance, and control protein science development from an artificial intelligence perspective. Our cross-functional team aims at accelerating the steps of translating the basic scientific knowledge from protein science laboratories into AI applications. Here, we report a comprehensive, balanced systematic review for the literature in the inter-field and a biochemical meta-analysis, which includes a classification of screened articles: *1*) by the ML techniques, they use and narrowing down the subareas, *2*) by the classes, methods, algorithms, prediction type and programming language, *3*) by some protein science queries, *4*) by protein science applications, and *5*) by protein science problems. Moreover, we present the main contributions of AI in several tasks, as well as a general outline of the processes that are carried out throughout the construction of the models and their applications. We outline a discussion on the best practices of validation, cross-validation, and individual control of testing ML models in order to assess the role that they play in the progress of ML techniques, integrating several data types and developing novel interpretations of computational methodology, thus enabling a wider range of protein’s-universe impacts. Finally, we provide future direction for machine learning approaches in the design of novel proteins, metabolic pathways, and synthetic redesign of protein networks.

## Materials and Methods

**FIGURE 1:**
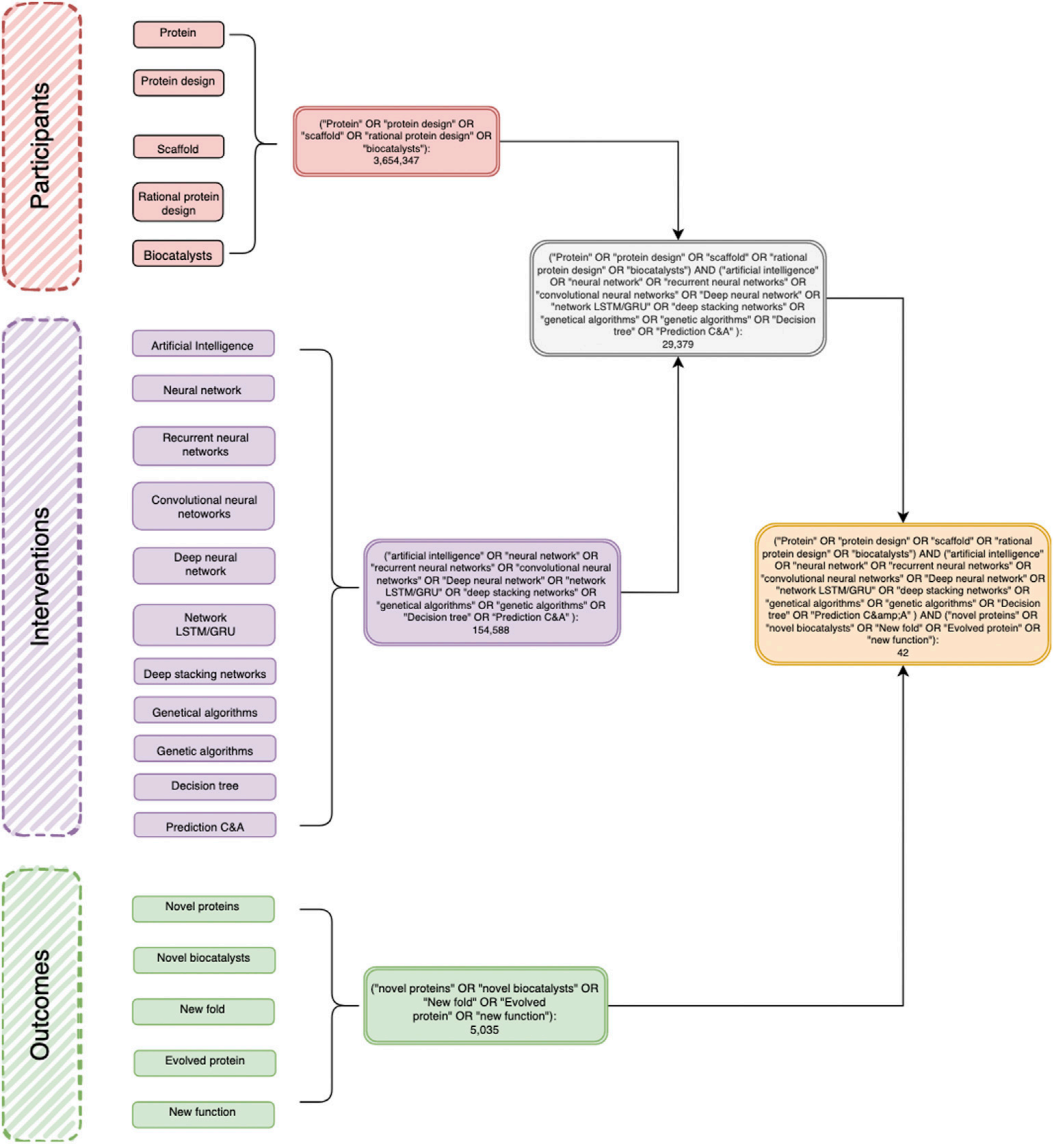
A representative decision diagram showing the articles retrieved using the PIO strategy in the PubMed database. P (participants): Protein, Protein Design, Scaffold, Rational protein design, Biocatalysts. I (intervention): Networks: Neural networks, Recurrent neural networks, Networks LSTM/GRU, Convolutional neural network, Deep belief networks, Deep stacking networks C5.0; Genetic algorithms; Artificial intelligence; Decision trees; Classification; Prediction C&A; Software: Weka, RapidMiner, IBM Modeler; Programming Languages: Python, Java, OpenGL, C++ Shell; Development platform: Caffe Deep Learning, TensorFlow, IBM Distributed Deep Learning (DDL); Paradigm: Supervised Learning, Unsupervised learning, Reinforced learning, new function.

**FIGURE 2:**
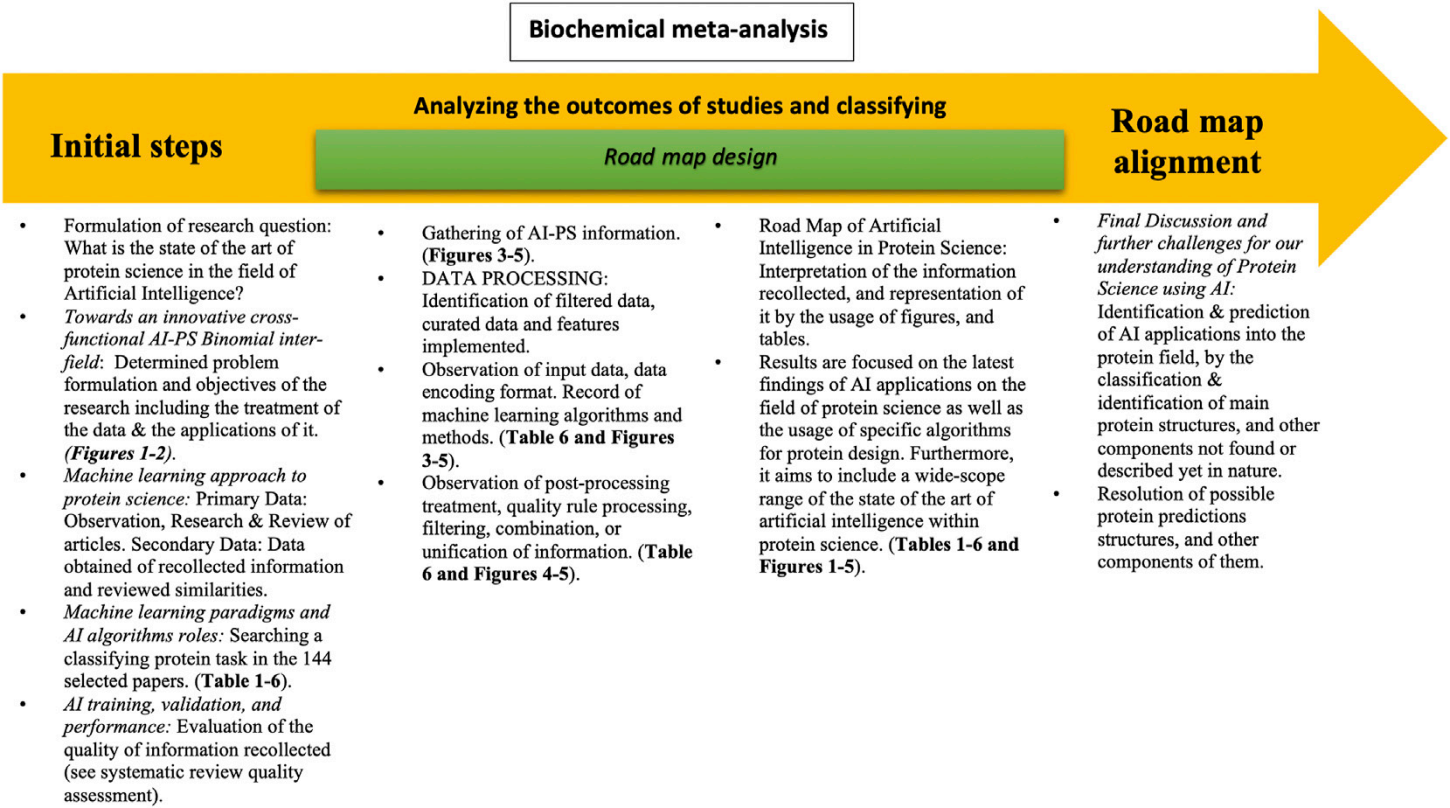
Flowchart of article scaffold. Representation of the process throughout the entire article. The biochemical meta-analysis consists of three main steps: the systematic review, the road map design, and the road map alignment. In the systematic review, the research question is formulated in order to set the basis and objectives of the project. It also includes the observation and synthesis of information obtained from a variety of articles and the correlation made between them. The latter followed by the quality evaluation of the collected information. The road map design consists of analyzing the outcome of the studies and classifying them, thus being able to interpret the information recollected and represent it through the usage of figures and tables. This aims to include a wide range of the state of the art or artificial intelligence. Finally, the road map alignment includes the final discussion and further changes for our understanding of protein science using AI and the resolution of possible protein science application targets.

**FIGURE 3:**
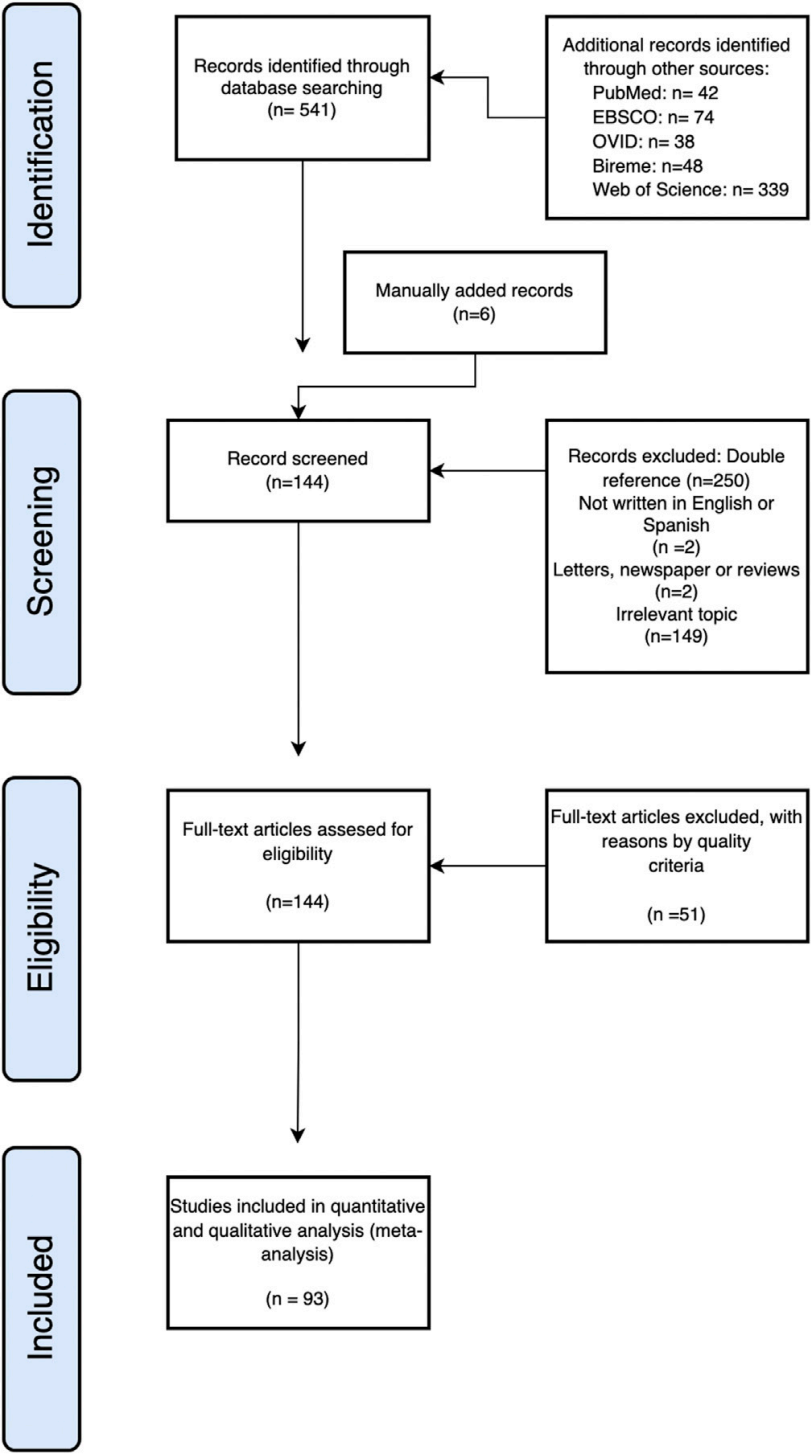
Flowchart of the review process. A PRISMA flowchart of the systematic review on AI for protein sciences.

A systematic review of the scientific literature found in the period (until February 2021) was carried out for this study (Figures 1–3) following the PIO (participants/intervention/outcome) approach and according to PRISMA declaration (Preferred Reporting Items for Systematic Reviews and Meta-Analyses) Supplementary. No ethical approval or letter of individual consent was required for this research.

### PIO Strategy

One of the main objectives is to discuss new information in the latest findings about the functions of AI in protein design. Furthermore, this review and meta-analysis intend to include a wide scope of the status of artificial intelligence in protein science. The PIO (participants, intervention, and outcome) strategy was used to systematically search all databases and was the methodology to address the following research questions: What is the state of art in the use of artificial intelligence in the protein science field? What is the use of neural networks in the rational design of proteins? Which neural networks are used in the rational design of proteins? Protein design is currently considered a challenge. As artificial intelligence makes progress, this is presented as a solution to various issues toward addressing how this new branch can be used for the creation of high precision models in protein design. Following the PIO strategy, the next terms were used for the research.

**Participants:** articles about proteins and their MeSH terms in general were considered for inclusion; we gave special consideration to protein design and their related terms such as scaffold (as a main structure or template), rational design, and biocatalysts (as a main task target for protein evolution and design in the chemical–biotechnological industry and biomedical field):

• protein
• protein design
• scaffold
• rational protein design
• biocatalysts

**Intervention:** studies with any types of algorithms, software, programming language, platform, or paradigm using alone or in combination were selected.

Types of algorithms:

• neural networks
• recurrent neural networks
• network LSTM/GRU
• convolutional neural network
• deep belief networks
• deep stacking networks C5.0
• genetic algorithms
• artificial intelligence
• decision trees
• classification
• prediction C&A

Software:

• Weka
• RapidMiner
• IBM Modeler

Programming languages:

• Python
• Java
• OpenGL
• C++
• Shell

Development platform:

• Caffe
• DeepLearning4j
• TensorFlow
• IBM distributed deep learning (DDL)

Paradigm:

• supervised learning
• unsupervised learning
• reinforced learning

Outcomes:

• novel proteins
• protein structure prediction
• novel biocatalysts
• new fold
• evolved protein
• new function

### Databases and Searches

The electronic databases used were PubMed, Bireme, EBSCO, and OVID. The concepts with similarity were searched with “OR,” and within the groups of each element of the PIO research, they were searched with the word “AND.” Next, a diagram was constructed in order to show the history of searches and concepts used (figure tree diagram). This figure describes in full detail the searching strategy in the PubMed database as well as all keywords used. Moreover, it includes the number of resulting articles. Subsequently, the results obtained from these searches were recorded. The references themselves were then downloaded into the Mendeley database. All references were taken, organized, and saved in *Mendeley*, eliminating duplicates for the final result.

### Biochemical Meta-analysis

The biochemical meta-analysis included formulating the research question, searching and classifying protein tasks in the 144 selected studies, gathering AI–PS information from the 144 studies, evaluating the quality of the studies (as described in the systematic review, see flowchart of PRISMA), analyzing and classifying the intervention and outcome of studies (networks, software, programming languages, development platforms, paradigms, novel proteins, novel scaffold, new fold, *etc*.), and building up tables and figures for the interpretation of evidence and presenting the results.

By a biochemical meta-analysis, we mean an analysis resulting from two processes: **identification** and **prediction**. The former consists of identifying AI applications into the protein field: classify and identify active and allosteric sites, molecular signatures, and molecular scaffolding not yet described in nature, each of which constitute a single part of a grand-type Lego structure. The latter is an analysis of resulting predictions: structures, molecular signatures, regulatory and ligand sites, *etc*.

### Biochemical Meta-Analysis and Designing the Road Map

PRELIMINARY: we determined the formulation of the problem and objectives of the research within the figure, which includes the treatment of the data and their applications. Note: the information was acquired from a list of various databases from which data were analyzed.

DATA COLLECTION: primary data: observation, research and review of articles. Secondary data: data of the reviewed articles and information shared among keywords.

DATA PRE-PROCESSING (ETL and training): identification of filtered data, curated data, and features implemented; machine learning input relationship with protein science servers.

DATA PROCESSING (training data and feature extraction): observation of input data and data encoding format. Record of machine learning algorithms and methods. Recognition of key information for processing data within databases.

DATA POST-PROCESSING: observation of post-processing treatment, rule quality processing, filtering, combination, or unification of information.

MEASURE: explanation of the process, the values of different metrics for the quantification of magnitudes, and the contribution for the completion within the process of information.

ANALYZE: identify the application of machine learning algorithm in which the input of the dataset to process data format, training set, and 3D structures.

IMPROVE: determine the set to whom these new forms will be applied in models of the researched data and contribute to future implementations in protein science.

Concerning the computational aspects as to how articles were classified, three initial divisions were made and are displayed in Table 1: Pre-process, process, and post-process, each of which contain, in turn, the following items:

**TABLE 1:** An overview of the included articles on study and algorithm features based in their characteristics, strengths, limitations, and measure of precision.

Author/Year of Publication/Setting	Classes of machine learning	Methods	Algorithms	Protein Query	Characteristics	Strengths	Limitations	Validation and performance
Study characteristics and algorithm aspects								
Song J., 2021, China (Song et al., 2021)	Connectionist and Symbolist	An ensemble predictor with a deep convolutional neural network and LightGBM with ensemble learning algorithm	CNN, LightGBM	A sequence-based prediction method for protein–ATP-binding residues, including, PSSM, the predicted secondary structure, and one-hot encoding	The CNN frameworks are proposed as a multi-incepResNet-based predictor architecture and a multi-Xception-based predictor architecture. LightGBM, as a Gradient Boosting Decision Tree (GBDT) for classification and regression merged by an ensemble learning algorithm	The model enriches the protein–ATP-binding residue prediction ability using sequence information. Outstanding performance using ensemble learning algorithm in combination with a deep convolutional neural network and LightGBM as an ATP-binding tool	Distribution of the specific weights was calculated according to the ratio between the positive instances and the negative instances to solve the imbalance problem. The sensitivity prediction was only 0.189. This can be attributed by its very limited prediction coverage and the limited number of sequences in the training set	AUC (0.922 and 0.902), MCC (0.639 and 0.0642), and 5-fold cross-validation
Verma N., 2021, US (Verma et al., 2021)	Connectionist	A DNN framework (Ssnet), for the protein–ligand interaction prediction, which utilize the secondary structure of proteins extracted as a 1D representation based on the curvature and torsion of the protein backbone	DNN	Information about locations in a protein where a ligand can bind, including binding sites, allosteric sites, and cryptic sites, independently of the conformation	Curvature and torsion of protein backbone, feature vector for ligand. Multiple convolution networks with varying window sizes as branch convolution	The model does not show biases in the physicochemical properties and necessity of accurate 3D conformation while requiring significantly less computing time. Fast computation once the model is trained with weights bare fixed. No requirement of high-resolution structural data	Ssnet being blind to conformation limits its capability to account for mutations resulting from the same fold but significant difference in binding affinity. Ssnet should be treated as a tool to cull millions of drug-like molecules and not as an exact binding affinity prediction tool	AUC, ROC, and EF scores
Bond. S, 2020, US (Bond et al., 2020)	Connectionist	CCP4i2 Buccaneer automated model-building pipeline	PDB	Correctness of protein residues	Visual examination by the crystallographer. Coot provides validation tools to identify Ramachandran outliers, unusual rotamers, and other potential errors, as well as an interface to some tools from MolProbity	No cutoff has to be chosen	It may also have difficulties in that a residue built into the solvent 5 A° away from the structure is no different than one 10 A° away	COD for main chain 0.751; COD for side chain 0.613
Kwon Y., 2020, Korea (Kwon et al., 2020)	Connectionist	A new neural network model for binding affinity prediction of a protein–ligand complex structure	3D-CNN	Protein–protein complexes in a 3D structure	Ensemble of multiple independently trained networks that consist of multiple channels of 3D CNN layers. Protein–ligand complexes were represented as 3D grids voxelized binding pocket and ligand	Higher Pearson coefficient (0.827) than the state-of-the-art binding affinity prediction scoring functions. Accurate ranking of the relative binding affinities of possible multiple binders of a protein, comparable to the other scoring functions	For docking power, the Ak-score-single model is not as prominent as the other criteria models	Spearman and Pearson correlation coefficients
Li H., 2020, France, Hong Kong (Hongjian et al., 2021)	Connectionist, Symbolist and Analogist	Analyzed machine learning scoring functions for structure-based virtual screening	RF, BRT, kNN, NN, SVM, GBDT, multi-task DNN XGBoost	Comparison and review of machine learning scoring functions and classical scoring functions	Machine learning-based scoring function performs better than classical scoring functions, outperforming the average classical methods	Machine learning-based scoring function has introduced strong improvements over classical scoring functions, benchmarks for SBVS.	Current SBVS benchmarks do not actually mimic real test sets, and thus their ability to anticipate prospective performance is uncertain	N/A
Liang M., 2020, China (Liang and Nie, 2020)	Connectionist	Method that uses the relation between amino acids directly to predict enzyme function	RN, LSTM	State description matrix containing structural information by four parts, amino acid name (N), angles φ and ψ(A), relative distance (RD), and relative angle γ (RA)	A three-layer MLP; a four-layer MLP; a three-layer MLP, all with ReLU nonlinearities. The final layer was a linear layer that produced logits for optimization with a softmax loss function	Structural relationship information of amino acids and the relationship inference model can achieve good results in the protein functional classification	The model is currently only for single-label classification rather than multi-label classification and only predicts proteins approximately into six major classes. The training has a considerable time during the entire experiment; further optimization is necessary to improve performance	Accuracy, ROC curve, AUC, 3-fold cross-validation
Nie J., 2020, Singapore, Taiwan (Sua et al., 2020)	Probabilistic inference, symbolist, and analogist	Identification of lysine PTM site from a convolutional neural network and sequence graph transform techniques	RF, SVM, MNB, LR, Max Entropy, KNN, CNN, MLP	A computational technique to improve the identification of reaction sites for multiple lysine PTM sites in a protein sample	Improves the performance of identifying lysine PTM sites by using a novel combination with convolutional neural networks and sequence graph transform	As the current model that we are proposing is a multilabel model, it is very generalizable, especially when it comes to combinations of multilabel that the dataset does not have. In addition, such combinations of multilabel will increase the test sample size and provide a better idea of the accuracy of the model	Deep learning models are black-box models and may not be very useful for trying to understand the causes of PTMs and how to affect them. We gather that scientists would like to know the cause and effect in order to propose disease modification methods, rather than just pure identification of PTM’s	Cross-validation, precision accuracy, recall, Hamming-loss
Qin Z., 2020, US (Qin et al., 2020)	Connectionist	Learn method on amino acid sequence folds into a protein structure, along with the phi–psi angle information for high resolution of protein structure	MNNN	Prediction with only primary amino acid sequence without any template or co-evolutional information	Performs labeling of dihedral angles, combined with the sequence information, allowing the phi–psi angle prediction and building the atomic structure	Prediction consumes less than six orders of magnitude time. Prediction of the structure of an unknown protein is achieved, showing great advantage in the rational design of *de novo* proteins	Prediction accuracy can be further improved by incorporating new structure to refine the model	Prediction accuracy (85%)
Savojardo C., 2020, Italy (Savojardo et al., 2020a)	Connectionist	A method for protein subcellular localization prediction	DeepMITO, 1D-CNN	Performing proteome-wide prediction of sub-mitochondrial localization on representative proteomes	Its major characteristics is to combine proteome-wide experimental data with the predicted annotation of subcellular localization at submitochondrial level and complementary functional characterization in terms of biological processes and molecular functions. Evolutionary information, in the form of Position-Specific Scoring Matrices (PSSM)	The model allows users to search for proteins by organisms, mitochondrial compartment, biological process, or molecular function and to quickly retrieve and download results in different formats, including JSON and CSV	N/A	MCC coefficient
Wang M., 2020, US (Wang M. et al., 2020a)	Symbolist	A topology-based network tree, constructed by integrating the topological representation and NetTree for predicting protein–protein interaction (PPI)	TopNetTree, CNN, GBT	Protein structures, protein mutation, and mutation type	Convolutional Neural Networks, used in their Top Net Tree model, as a second module: consisting of the CNN-assisted GBT model	The proposed model achieved significantly better Rp than those of other existing methods, indicating that the topology-based machine learning methods have a better predictive power for PPI systems	Both GBTs and neural networks are quite sensitive to system errors of training of a model The ΔΔG of 27 non-binders (–8 kcal mol–1) did not follow the distribution of the whole dataset.	Person coefficient (Rp) = 0.65/0.68 and 10-fold cross-validation
Wardah W., 2020, Australia, Fiji, Japan, US (Wardah et al., 2020)	Pattern recognition	A convolutional neural network to identify the peptide-binding sites in proteins	CNN	Amino acid residues to create the image-like representations by feature vectors	Sets of convolution layers for image operations, followed by a pooling layer and a fully connected layer. The internal weights of the network were adjusted using the Adam optimizer. Bayesian optimization uses calculated values for configuring the model’s hyper-parameters based on prior observations	The model is able to predict a protein sequence with the highest sensitivity compared to any other tool	Improvement and especially in reducing the number of non-binding residues that get falsely classified as binding sites. Better feature engineering to produce better protein–peptide-binding site prediction results. More advanced computing environment	Sensitivity, specificity, AUC, ROC curve, and MCC coefficient
Yu C., 2020, Taiwan, US (Yu and Buehler, 2020)	Connectionist	A deep neural network model is based on translating protein sequences and structural information into a musical score, reflecting secondary structure information and information about the chain length and different protein molecules	RNN, LSTM	A vibrational spectrum of the amino acid, comprising amino acid sequence, fold geometry, or secondary structure	The RNN layers, Long Short-Term Memory Units are for time sequence features, alongside a dynamical conditioning. The attention dynamical conditioning model monitors the note velocity changes of the note sequences	The deep neural network is capable of training, classifying, and generating new protein sequences, reproducing existing sequences, and completely new sequences that do not exist yet. The model generates new proteins with an embedded secondary structure approach	The method could be extended to address folded structures of proteins by including more spatial information (relative distance of residuals, angles, or contact information). As well as the addition of combined optimization algorithms, like genetic algorithms	Molecular dynamics equilibration with normal mode analysis
Cui Y., 2019, China (Cui et al., 2019)	Pattern recognition	A deep learning model sequence-based for ab initio protein–ligand-binding residue prediction	DCNN	Protein sequences in order to construct several features for the input feature map	First representation, an amino acid sequence by m x d. First convolutional layer with k x d kernel size. Stage 1, with Plain(k x 1,2c) the same as for Block(k x 1,2c). Stage 2, with a Block(k x 1,2c) and Layer normalization-GLU-Conv block	The convolutional architecture provides the ability to process variable-length inputs. The hierarchical structure of the architecture enables us to capture long-distance dependencies between the residue and those that are precisely controlled. Augmentation of the training sets slightly improves the performance	The computational cost for training increases several times. Due to the considerable data skew, the training algorithm tends to fall into a local minimum where the network predicts all inputs as negative examples	Precision, Recall, MCC
Degiacomi M., 2019, UK (Degiacomi, 2019)	Deep machine learning	Conformational space generator	Molecular dynamics, random forests and autoencoder algorithms	Generative neural network trained on protein structures produced by molecular simulation can generate plausible conformations	Generative neural networks for the characterization of the conformational space of proteins featuring domain-level dynamics	The auto encoder does great at describing concerted motions (e.g., hinge motions) than at capturing subtle local fluctuations; it is most suitable to handle cases featuring domain-level rearrangements	This generative neural network model yet lies incapable of reproducing non-diversity-related cases, which is a subject of active research in the machine learning community	Performance assessed using different sizes of latent vector and optimizer
Fang C., 2019, China, Japan (Fang et al., 2019)	Connectionist	Protein sequence descriptor, position-specific scoring matrix, en DCNNMoRF	DCNN	Pinpoint molecular recognition features, which are key regions of intrinsically disordered proteins by machine learning methods	Ensemble deep convolutional neural network-based molecular recognition feature prediction. It does not incorporate any predicted features from other classifiers	The proposed method is highly performant for proteome-wide MoRF prediction without any protein category bias	It is yet difficult to predict if the new models will perform better only on the results, referring to the use of a new dataset.	Sensitivity, Specificity, Accuracy, AUC, ROC curve, MCC coefficient
Fang C., 2019, US (Fang et al., 2020)	Connectionist	Deep dense inception network for beta-turn prediction	DeepDIN	Protein sequence by creating four sets of features: physicochemical, HHBlits, predicted shape string and predicted eight-state secondary structure	Concatenate four convolved feature maps along the feature dimension. Feed the concatenated feature map into the stringed dense inception blocks. Dense layer, with Softmax function	Proposed process for beta-turn prediction outperforms the previous authors	Of the nine cases used, the amount of data belonging to each class may not produce a model with the ability to extract features or to be well generalized. Combined features improve prediction results than those features used alone	MCC and 5-fold cross-validation
Fu H., 2019, China (Fu et al., 2019)	Analogist	Classification Natural language prediction (NLP) task	CNN DL	Predict Lysine ubiquitination sites in large-scale	Input fragment. Multi-convolution-pooling layers. Fully connected layers	Extract features from the original protein fragments. First used in the prediction of ubiquitination	DeepUbi is not too deep. Only two convolution-pooling structures	4-, 6-, 8-, and 10-fold cross-validation Sensitivity, Specificity, Acc, AUC, MCC, Acc >85% AUC = 0.9066/MCC= 0.78
Guo Y., 2019, US (Guo et al., 2019)	Connectionist and Symbolist	Asymmetric Convolutional neural networks and bidirectional long short-term memory	ACNNs, BLSTM, DeepACLSTM	Sequence-based prediction for Protein Secondary Structure (P.S.S.)	The DeepACLSTM method is proposed to predict an 8-category PSS from protein sequence features and profile features	The method efficiently combines ACNN with BLSTM neural networks for the PPS prediction. Leveraging the feature vector dimension of the protein feature matrix	Expensive and time consuming	CB6133 0.742 CB513 0.705
Haberal I., 2019, Norway, Turkey (Haberal and Ogul, 2019)	Connectionist	Three different deep learning architectures for prediction of metal-binding of Histidine (HIS) and Cysteine (CYS) amino acids	2D CNN, LSTM, RNN	Three methods, PAM, ProCos, and BR to create the feature set from the frame vector; applying directly to raw protein sequences without any extensive feature engineering, while optimizing the model for predicting metal-binding site	The model is a 2D-CNN with four convolution layers, two pooling, two dropout, and two multi-layer perceptron layers. Each convolution layer has 3 × 3 pixel filters	The good performance of the model demonstrates the potential application for protein metal-binding site prediction. A competitive tool for future metal-binding studies, protein metal -interaction, protein secondary structure prediction, and protein function prediction. The CNN method provides better results for the prediction of protein metal binding using PAM attributes	The overall best results were obtained for a window of size 15. The lowest result was obtained in windows of size 101. The lowest result for the ProCos was obtained with the CNN model	Precision, Accuracy, Recall F-Measures K-fold (k = 3,5) cross-validation
Heinzinger M., 2019, Germany (Heinzinger et al., 2019)	Connectionist	Natural language processing with Deep learning	ELMo CharCNN LSTM	Protein function and structure prediction *via* analysis of unlabeled big data and deep learning processing	Novel representation of protein sequences as continuous vectors using language model ELMo, using NLP.	The approach improved over some popular methods using evolutionary information, and for some proteins even did beat the best. Thus, they prove to condense the underlying principles of protein sequences. Overall, the important novelty is speed	Although SeqVec embeddings generated the best predictions from single sequences, no solution improved over the best existing method using evolutionary information	Predictions of intrinsic disorder were evaluated through Matthew’s correlation coefficient and the False- Positive Rate. Also, the Gorodkin measure was used
Kaleel M., 2019, Ireland (Kaleel et al., 2019)	Connectionist and Symbolist	Deep neural network architecture composed of stacks of bidirectional recurrent neural networks and convolutional layers	RSA.	Three-dimensional structure of protein prediction	Predicting relative solvent accessibility (RSA) of amino acids within a protein is a significant step toward resolving the protein structure prediction challenge, especially in cases in which structural information about a protein is not available by homology transfer	High accuracy in four different classes (75% average). They performed all the training and testing in 5-fold cross-validation on a very large, state-of-the-art redundancy reduced set containing over 15,000 experimentally resolved proteins	The protein structure prediction challenge especially in cases in which structural information about a protein is not available by homology transfer	2-class ACC 0.805 2-class F1 0.80 3-class ACC 0.664 3-class F1 0.66 4-class ACC 0.565 4-class F1 0.56
Karimi M., 2019, US (Karimi et al., 2019)	Pattern recognition	Interpretable deep learning of compound–protein affinity	RNN–CNN models	Development of accurate deep learning models for predicting compound–protein affinity using only compound identities and protein sequences	Using only compound identities and protein sequences, and taking massive protein and compound data, RNN–CNN, and GCNN trained models outperform baseline models	Compared to conventional compound or protein representations using molecular descriptors or Pfam domains, the encoded representations learned from novel structurally annotated SPS sequences and SMILES strings improve both predictive power and training efficiency for various machine learning models	The resulting unified RNN/GCNN–CNN model did not improve against unified RNNCNN	Inferior relative error in IC50 within 5-fold for test cases and 20-fold for protein classes not included for training
Li C., 2019, China (Li and Liu, 2020)	Constrained optimization and Connectionist	Feature extractor techniques for protein-fold recognition	MotifCNN and MotifDCNN SVM CNN	Fold-specific features with biological attributes considering the evolutionary information from position-specific frequency matrices (PSFMs) considering the structure information from residue–residue	The predictor called MotifCNN-fold combines SVMs with the pairwise sequence similarity scores based on fold-specific features	The model incorporates the structural motifs into the CNNs, aiming to extract the more discriminative fold-specific features with biological attributes, considering the evolutionary information from PSFMs and the structure information from CCMs	Existing fold-specific features lack biological evidences and interpretability, the feature extraction method is still the bottleneck for the performance improvement of the machine learning-based methods	2-fold cross-validation, Accuracy
Lin J., 2019, China (Lin et al., 2019)	Analogist and evolving structures	A drug target prediction method based on genetic algorithm and Bagging-SVM ensemble classifier	GA, SVM	Protein sequences by combining pseudo amino acid, dipeptide composition, and reduced sequence algorithms	GA is used to select the druggable protein dataset. The optimal feature vectors are for the SVM classifier. Bagging-SVM ensemble is for positive and negative sample sets	The method has a high reference value for the prediction of potential drug targets. An improvement over previous methods	N/A	Acc, MCC, Sn, Sp, AUC, PPV, NPV, F1-score,ROC curve and 5-fold cross-validation
Pagès G., 2019, France (Pagès et al., 2019)	Connectionist	Regression structure atomic depiction with a density function	3D CNN	Protein model quality assessment	Three convolutional layers. Fully connected layers. Use of ELU as activation function	Competitivity with single-model protein model quality assessment. Trained to match CAD-score, on stage 2 of CASP 11	Ornate does not reach the accuracy of the best meta-methods. Scoring time about 1 s for mid-size proteins	Network running using a GeForce GTX 680 GPU
Picart-Armada S., 2019, Belgium, UK, Spain (Picart-Armada et al., 2019)	Pattern recognition	Network propagation machine learning methods	PR, Random Randomraw EGAD, PPR, Raw, GM, MC, Z-scores, KNN, WSLD, COSNet, bagSVM, RF, SVM	Assess performance of several network propagation algorithms to find sensible gene targets for 22 common non-cancerous diseases	Two biological networks, six performance metrics, and compared two types of input gene-disease association scores. The impact of the design factors in performance was quantified through additive explanatory models	Network propagation seems effective for drug target discovery, reflecting the fact that drug targets tend to cluster within the network	Choice of the input network and the seed scores on the genes need careful consideration due to possibility of overestimation in performance indicators	There was a dramatic reduction in performance for most methods when using a complex-aware cross-validation strategy. Three cross-validation schemes were used
Savojardo C., 2019, Italy (Savojardo et al., 2020b)	Connectionist	A convolutional neural network architecture to extract relevant patterns from primary features	CNN	High prediction on discriminating four mitochondrial compartments (matrix, outer, inner, intermembrane)	Two pooling layers concatenated into a single vector with four independent output units with sigmoid activation function quantifying the membership of each considered mitochondrial compartment	Model has a robust approach with respect to class imbalance and accurate predictions for the four classification compartments	Adoption of more complex architecture, like recurrent layers can improve performance. However, the use of recurrent models leads to bad performance. Impossibility to predict multiple localization for a single protein sequence	10-fold cross-validation, MCC from 0.45 to 0.65
Schantz M., 2019, Argentina, Denmark, Malaysia (Klausen et al., 2019)	Connectionist	NetSurfP-2.0	NetSurfP-2.0	Predict local structural features of a protein from the primary sequence	A novel tool that can predict the most important local structural features with unprecedented accuracy and runtime. Is sequence-based and uses an architecture composed of convolutional and long short-term memory neural networks trained on solved protein structures.	Predicts solvent accessibility, secondary structure, structural disorder, and backbone dihedral angles for each residue of the input sequences	The models are presented with cases that are neither physically nor biologically meaningful	CASP12 0.726 TS115 0.778 CB513 0.794
Taherzadeh G., 2019, Australia, US (Taherzadeh et al., 2019)	Constrained optimization and Connectionist	Predictor method of N- and mucin-type O-linked glycosylation sites in mammalian glycoproteins	DNN, SVM	An amino acid sequence binary vector, evolutionary information, physicochemical properties	DNN uses deep architectures of fully connected artificial neural networks. And SVM linear kernel for classification techniques to predict O-linked glycosylation sites	N-glycosylation model performs equally well for intra or cross-species datasets	Limitation to typical N-linked and mucin-type O-linked glycosylation sites due to lack of data for atypical N-linked and other types of O-linked glycosylation sites	AUC MCC, accuracy, sensitivity, specificity, ROC curve, 10-fold cross-validation
Torng W., 2019, US (Torng and Altman, 2019)	Analogist	Classification Softmax classifier for class probabilities	3D CNN SVM	Protein functional site detection	Protein site representation as four atom channels and supervised labels	Achieved an average of 0.955 at a threshold of 0.99 on PROSITE families. Good performance where sequence motifs are absent, but a function is known	Loss of specific orientation data. NOS structures 1TLL and 1F20 and catalytic sites in TRYPSIN-like enzymes not detected	5-fold cross-validation Precision, Recall Precision = 0.99 Recall = 0.955
Wan C., 2019, UK (Wan et al., 2019)	Connectionist	A novel method (STRING2GO), with a deep maxout neural networks for protein functional predictive information	DMNN, SVM	Protein functional biological network node neighborhoods and co-occurrence function information	The network architecture consists of three fully connected hidden layers, followed by an output layer with as many neurons as the numbers of terms selected for the biological process functional domain. A sigmoid function is used as activation function and the AdaGrad optimizer is implemented	Successful learning of the functional representation classifiers for making predictions	Potential improvement of predictive accuracy by integrating representations from other data sources with the current PPI network embedding representations	AUC, ROC, MCC
Wang D., 2019, China (Wang D. et al., 2020)	Evolutionary	An Artificial Intelligence-based protein structure Refinement method	Multi-objective PSO	Query sequence structures as the initial particle selection for conformation representation	Use of multiple energy functions as multi-objectives. Initialization, energy map of the initial particles. Iteration, energy landscape of the 4th iteration. Selection of non-dominated solutions and added to the Pareto set. And selection of the global best position and the best position every swarm has had by the use of the dominance relationship of swarms, moving to the optimal direction	Success of AIR can be attributed to three main aspects: the first is the anisotropy of multiple templates. The complementarity of multi-objective energy functions and the swarm intelligence of the PSO algorithm, for effective search of good solutions. The larger number of iterations allows the algorithm to perform a more detailed search on the search space, which can improve the quality of the output models	Restriction of the velocity of the dihedral angles in each iteration to a reasonable range for balancing the accuracy and the searching conformation. There are still some unreasonable solutions in the Pareto set. The final step, which ranks the structures in Pareto set, needs more studies	RMSD value
Yu C., 2019, US (Yu et al., 2019)	Connectionist	Regression musical patterns by the extension of protein designed	RNN LSTM	Generation of audible sound from amino acid sequence for application on designer materials	An RNN utilized for melody generation. (LSTM) for time sequence featuring	Mechanism to explain the importance of protein sequences. 4.- It can be applied to express the structure of other nanostructures	N/A	N/A
Zhang D., 2019, US (Zhang and Kabuka, 2019)	Connectionist	Protein sequence pre-processing, unsupervised learning, supervised, and deep feature extraction	Multimodal DNN	Identify protein–protein interactions and classify families *via* deep learning models	Multi-modal deep representation learning structure by incorporating the protein physicochemical features with the graph topological features from the PPI networks	The model outperforms most of the baseline machine learning models analyzed by the authors, using the same reference datasets	If there is a certain type of PPI that previous models cannot handle, the article will not say if the new model can	PPI prediction accuracy for eight species ranged from 96.76 to 99.77%, which implies the multi-modal deep representation-learning framework achieves superior performance compared to other computational methods
Zhang Y., 2019, China (Zhang et al., 2019)	Connectionist	A new prediction approach appropriate for imbalanced DNA–protein-binding sites data	ADASYN	Employment of PSSM feature and sequence feature for predicting DNA-binding sites in proteins	Introduction of new feature representation approach by combining position-specific scoring matrix, one-hot encoding and predicted solvent accessibility features. Apply adaptive synthetic sampling to oversample the minority class and Bootstrap strategy for a majority class to deal with the imbalance problem	Demonstration that the method achieves a high prediction performance and outperforms the state-of-the-art sequence-based DNA–protein-binding site predictors	Consideration of some other physicochemical features to construct the model and try to explain the biological meaning of CNN filters	Sensitivity, Specificity, Accuracy, Precision, and MCC coefficient
Zheng W., 2019, US (Zheng et al., 2019)	Probabilistic inference, Symbolist	Two fully deep learning automated structure prediction pipelines for guided protein structure prediction	Zhang-Server and QUARK	Starting from a full-length query sequence structure	Three core modules: multiple sequence alignment (MSA) generation protocol to construct deep sequence-profiles for contact prediction; an improved meta- method, NeBcon, which combines multiple contact predictors, including ResPRE that predicts contact-maps by coupling precision-matrices with deep residual convolutional neural networks; an optimized contact potential to guide structure assembly simulations	Improvement of the accuracy of protein structure prediction for both FM and TBM targets. Accurate evolutionary coupling information for contact prediction, thus improving the performance of structure prediction. And properly balancing the components of the energy function was vital for accurate structure prediction	Incorrect prediction of contacts between the N- and C- terminal protein regions. Low accuracy of contact prediction in the Terminal regions due to MSAs with many gaps in these regions, as the accuracy of contact-map prediction and FM target modeling is highly influenced by the number of effective sequences in the MSA.	TM-score and *p*-values
Cuperus J., 2018, US (Cuperus et al., 2017)	Connectionist	Regression dropout probability distribution	DNN, CNN, LSTM	Predict protein expression	Hierarchical representation of image features from data	Prediction and visualization of transcription factor binding, Dnase I hypersensitivity sites, enhancers, and DNA methylation sites	Measurement of protein expression with yeast possessing only 5000 genes	k-mer feature, Cross-validation, Held-out R2 = 0.61
Fang C.,US, 2018 (Fang et al., 2018)	Pattern recognition	A deep learning network architecture for both local and global interactions between amino acids for secondary structure prediction	Deep3I	A protein secondary structure prediction model	A designed feature matrix corresponding to the primary amino acid sequence of a protein, which consists of a rich set of information derived from individual amino acid, as well as the context of the protein sequence	This model uses a more sophisticated, yet efficient, deep learning architecture. The model utilizes hierarchical deep inception blocks to effectively process local and nonlocal interactions of residues	Further application of the model to predict other protein structure-related properties, such as backbone torsion angles, solvent accessibility, contact number, and protein order/disorder region, will be done in the future	Accuracy, *p*-value
Feinberg E., 2018, China, US (Feinberg et al., 2018)	Connectionist	A PotentialNet family of graph convolutions	GCNN	A generalized graph convolution to include intramolecular interactions and noncovalent interactions between different molecules	First: graph convolutions over only bonds, which derives new node feature maps. Second: entails both bond-based and spatial distance-based propagations of information. Third: a graph gather operation is conducted over the ligand atoms, whose feature maps are derived from bonded ligand information and spatial proximity to protein atoms	Statistically significant performance increases were observed for all three prediction tasks, electronic property (multitask), solubility (single task), and toxicity prediction (multitask). Spatial graph convolutions can learn an accurate mapping of protein−ligand structures to binding free energies using the same relatively low amount of data	Drawback to train−test split is possible overfitting to the test set through hyperparameter searching. Another limitation is that train and test sets will contain similar examples	Regression enrichment factor (EF), Pearson, and Spearman coefficient, R-squared, MUE (mean-unsigned error)
Frasca M., 2018, Italia (Frasca et al., 2018)	Analogist	Clustering Hopfield model	COSNet ParCOSNet HNN	AFP (Automated Protein Function Prediction)	Network parameters are learned to cope with the label imbalance	Advantage of the sparsity of input graphs and the scarcity of positive proteins in characterizing data in the AFP.	Time execution increased less than the density, and more than the number of nodes	5-fold cross-validation Implementation and execution in a Nvidia GeForce GTX980 GPU target Precision, Recall, F-score, AUPRC
Hanson J., Australia, China, 2018 (Hanson et al., 2019)	Pattern recognition	A sequence-based prediction of one- dimensional structural properties of proteins	CNN, LSTM-BRNN	Improving the prediction of protein secondary structure, backbone angles, solvent accessibility	The model leverages an ensemble of LSTM-BRNN and ResNet models, together with predicted residue–residue contact maps, to continue the push toward the attainable limit of prediction for 3- and 8-state secondary structures, backbone angles (h, s, and w), half-sphere exposure, contact numbers and solvent accessible surface area (ASA)	The large improvement of fragment structural accuracy. A new method for predicting one-dimensional structural properties of proteins based on an ensemble of different types of neural networks (LSTM-BRNN, ResNet, and FC-NN) with predicted contact map input from SPOT-contact. The employment of an ensemble of different types of neural networks contributes another 0.5% improvement	Long proteins are also shown to take extensive time, especially for 2D analysis tools. The use of CPU and GPU is shown to not make a major difference in the time taken, as the speed increase introduced by GPU acceleration mainly comes during training	10-fold cross-validation, Accuracy
Hanson J., Australia, China, 2018 (Hanson et al., 2018)	Connectionist	Method by stacking residual 2D-CNN with residual bidirectional recurrent LSTM networks, with 2D evolutionary coupling-based information	CNN, 2D-BRLSTM	Protein contact map prediction	Transformation of sequence-based 1D features into a 2D representation (outer concatenation function). ResNet, 2D-BRLSTM and FullyConnected (FC)	Method achieves a robust performance. The model is more accurate in contact prediction across different sequence separations, proteins with a different number of homologous sequences and residues with a different number of contacts	Coding limitation environment imposed by the 2D-BRLSTM model; training and testing input is limited to proteins of length 300 and 700 residues	AUC >0.95, ROC curve, precision
Huang L., 2018, US (Huang et al., 2008)	Connectionist	A novel PPI prediction method based on deep learning neural network and regularized Laplacian kernel	ENN-RL	Protein–protein interaction network	Contains five layers including the input layer, three hidden layers, and the output layer. Sigmoid is adopted as the activation function for each neuron, and layers are connected with dropouts. Regularized Laplacian kernel applied to the transition matrix built upon that evolved the PPI network	The transition matrix learned from our evolution neural network can also help build optimized kernel fusion, which effectively overcome the limitation of the traditional WOLP method that needs a relatively large and connected training network to obtain the optimal weights	The results show that our method can further improve the prediction performance by up to 2%, which is very close to an upper bound that is obtained by an approximate Bayesian computation-based sampling method	Cross-validation, AUC, sensitivity
Khurana S., 2018,Qatar, USA (Khurana et al., 2018)	Analogist	Clustering Natural language processing task	CNN FFNN	Solubility prediction	Use additional biological features from external feature extraction tool kits from the protein sequences	DeepSol is at least 3.5% more accurate than PaRSnIP and 15% than PROSO II. DeepSol is superior to all the current sequence-based protein solubility predictors	DeepSol S2 model was surpassed by PaRSnIP on sensitivity for soluble proteins	10-fold cross-validation Acc, MCC 15% MCC = 0.55 3.5% DeepSol S1- 69 DeepSol S2- 69%
Le N., 2018, Taiwan (Le et al., 2018)	Analogist	Regression Softmax layer for classification	CNN	Classify Rab protein molecules	2D-CNN and position-specific scoring matrices. PSSM profiles of 20 × 20 matrices	Construct a robust deep neural network for classifying each of four specific molecular functions. Powerful model for discovering new proteins that belong to Rab molecular functions	Consideration of the potential effects of more rigorous classification tests	5-fold cross-validation Sensitivity, Specificity, Acc, AUC, F-score, MCC Acc = 99, 99.5, 96.3, 97.6%
Li H., 2018, China (Huang et al., 2018)	Constrained optimization	Regression Adam optimizer	DNN CNN LSTM	Prediction of protein interactions	Machine learning approach for computational methods for the prediction of PPIs	Insight into the identification of protein–protein interactions (PPIs) into protein functions	Manual input of features into the networks	Hold-out testing set model validation Acc, recall, precision, F-score, MCC Acc = 0.9878 Recall= 0.9891 Precision = 0.9861 F-score= 0.9876 MCC= 0.9757
Long H., 2018, China, US (Long et al., 2018)	Connectionist	Classification sigmoid function	HDL CNN LSTM RNN	Predicting hydroxylation sites	CNN deep learning model. Convolution layer consists of a set of filters through dimensions of input data	*p*-values between AUCs of other methods are less than 0.000001	Comparative results for CNN and iHyd-PseCp networks	5-fold cross-validation Sn, Sp, Acc, MCC, TPR, FPR, Precision, recall
Makrodimitris S., 2018, Netherlands (Makrodimitris et al., 2019)	Analogist	Clustering constrained optimization	KNN LSDR	Protein function prediction	Transformation of the GO terms into a lower-dimensional space	GO-aware LSDR has slightly better performance on SDp. LSDR reduces the number of dimensions in the label-space. Improve power of the term-specific predictors	LSDR generates inconsistent parent–child pairs. GO-aware terms have a higher inconsistencies	3-fold cross-validation Fp, AUPRCp, SDp, Ft, AUCRPCt
Popova M., 2018, Russia, US (Popova et al., 2018)	Constrained optimization	Regression Stack-RNN as a generative model	Stack-RNN LSTM.	*De novo* drug design	Deep neural network generative novel molecules (G) and predictive novel compounds (P)	The ReLeaSe method does not rely on predefined chemical descriptors No manual feature engineering for input representation	Extension of the system to afford multi-objective optimization of several target properties	5-fold cross-validation (5CV) model trained using a GPU Acc R2, RMSE Acc R2 = 0.91 RMSE = 0.53
Sunseri J., 2018, US (Sunseri et al., 2019)	Connectionist	Regression distributed atom densities	CNN	Cathepsin S model ligand protein	CNN based on scoring functions	CNN scoring function outperforms Vina on most tasks without manual intervention	Difficulties with Cathepsin S, for *de novo* docking	AUC, ROC, MCC
Zhang B., 2018, China (Zhang B. et al., 2018)	Connectionist	A novel deep learning architecture to improve synergy protein secondary structure prediction	CNN, RNN, BRNN	Four input features; position-specific scoring matrix, protein coding features, physical properties, characterization of protein sequence	A local block comprising two 1D convolutional networks with 100 kernels, and the concatenation of their outputs. BGRU block, the concatenation of input from the previous layer and before the previous layer is fed to the 1D convolutional filter. After reducing the dimensionality, the 500-dimensional data are transferred to the next BGRU layer	The CNN was successful at feature extraction, and the RNN was successful at sequence processing. The residual network connected the interval BGRU network to improve modeling long-range dependencies. When the staked layers were increased to two layers, the performance increased to 70.5%, and three-layer networks increased further to 71.4% accuracy	When the recurrent neural network was constructed by unidirectional GRU, the performance dropped to 67.2%. The unidirectional GRU network was ineffective at capturing contextual dependencies	Precision, Recall, F1-score, macro-F1, Accuracy
Zhang L., 2018, China (Zhang L. et al., 2018)	Connectionist	Two novel approaches that separately generate reliable noninteracting pairs, based on sequence similarity and on random walk in the PPI network	DNN, Adam algorithm	Use of auto-covariance descriptor to extract the features from amino acid sequences and deep neural networks to predict PPIs	The feature vectors of two individual proteins extracted by AC are employed as the inputs for these two DNNs, respectively. Adam algorithm is applied to speed up training. The dropout technique is employed to avoid overfitting. The ReLU activation function and cross-entropy loss are employed, since they can both accelerate the model training and obtain better prediction results	To reduce the bias and enhance the generalization ability of the generated negative dataset, these two strategies separately adjust the degree of the non-interacting proteins and approximate the degree to that of the positive dataset.	NIP-SS is competent on all datasets and hold a good performance, whereas NIP-RW can only obtain a good performance on small dataset (positive samples ≤6000) because of the restriction of random walk and the results of extensive experiments	Precision, Accuracy, Recall, Specificity, MCC coefficient, F1-score, AUC, Sensitivity
Zhao X., 2018, China (Zhao et al., 2018)	Connectionist	Bi-modal deep architecture with sub-nets handling two parts (raw protein sequence and physicochemical properties)	CNN and DNN	Raw sequence and physicochemical properties of protein for characterization of the acetylated fragments	Multi-layer 1D CNN for feature extractor and DNN with attention layer with a softmax layer	Capability of transfer learning for species-specific model, combining raw protein sequence and physicochemical information	Interpretation of biological aspect, overfitting problems on small-scale data	10-fold cross-validation; ACC = 0.708, sensitivity (SEN) = 0.723, specificity (SPE) = 0.707, AUC = 0.783, MCC = 0.251
Armenteros J., 2017, Denmark (Almagro Armenteros et al., 2017)	Analogist	Classification optimization	CNN RNN BLSTM FFNN Attention models	Predict protein subcellular localization	CNN extracts motif information using different motif sizes. Recurrent neural network scans the sequence in both directions	A-BLSTM and the CONV A-BLSTM models achieved the highest performance	Training time for the full ensemble was 80 h, approximately 5 h per model	Nested cross-validation and held-out set for testing models Gorodkin, Acc, MCC 72.90% 72.89%
Jimenez J., 2017, Spain (Jiménez et al., 2017)	Bayesian	Regression sigmoid activation function, depicting the probability	3D CNN	Predict protein–ligand-binding sites Drug design	Fully connected networks. Hierarchical organized layers	Four convolutional layers with max pooling and dropout after every two convolutional layers, followed by one regular fully connected layer	Demand of significant computational resources than other methods for ligand-binding prediction	10-fold cross-validation Using Nvidia GeForce GTX 1080 GPU for accelerated computing DCC, DVO AUC, ROC, Sn, SP, Precision, F1-score, MCC, Cohen’s Kappa coefficient
Müller A., 2017, Switzerland (Müller et al., 2018)	Analogist	Regression SoftMax function for temperature-controlled probability	RNN LSTM	Design of new peptide combinatorial *de novo* peptide design	The computed output y is compared to the actual amino acid to calculate the categorical cross-entropy loss	The network models were shown to generate peptide libraries of a desired size within the applicability domain of the model	Increasing the network size to more than two layers with 256 neurons led to rapid over-fitting of the training data distribution	5-fold cross-validation Network training and generated sequences on a Nvidia GeForce GTX 1080 Ti GPU
Ragoza M., 2017, US (Ragoza et al., 2017)	Connectionist	Classification distributed atom densities	CNN SGD	Protein-ligand score for drug discovery	CNN architecture: construction using simple parameterization and serve as a starting point for optimization	On a per-target basis, CNN scoring outperforms Vina scoring for 90% of the DUD-E targets	CNN performance is worse at intra-target pose ranking, which is more relevant to molecular docking	3-fold cross-validation ROC, AUC, FPR, TPR, RF-score, NNScore. CNN-0.815 Vina-0.645
Szalkai B., 2017, Hungary (Szalkai and Grolmusz, 2018a)	Pattern recognition	A classification by amino acid sequence multi-label classification ability	ANN	Protein classification by amino acid sequence	The convolutional architecture with 1D spatial pyramid pooling and fully connected layers. The network has six one-dimensional convolution layers with kernel sizes [6,6,5,5,5,5] and depths (filter counts) [128,128,256,256, 512,512], with parametric rectified linear unit activation. Each max pooling layer was followed by a batch normalization layer	The model outperformed the existing solutions and have attained a near 100% of accuracy in multi-label, multi-family classification	Network variants without batch normalization and five (instead of six) layers showed a performance drop of several percentage points. With more GPU RAM available, one can further improve upon the performance of our neural network by simply increasing the number of convolutional or fully connected layers	Precision, Recall, F1-value, AUC, ROC curve
Szalkai B., 2017, Hungary (Szalkai and Grolmusz, 2018b)	Logical Inference	Classification Hierarchical classification tree	ANN	Hierarchical biological sequence classification	SECLAF implements a multi-label binary cross-entropy classification loss on the output neurons	SECLAF produces the most accurate artificial neural network for residue sequence classification to date	Preparation of the input data must be done by the user	AUC
Vang Y., 2017, US (Vang and Xie, 2017)	Analogist	Regression Distributed representation with NLP	CNN	HLA class I-peptide-binding prediction	The CNN architecture: convolutional and fully connected dense layers	Effective for validation, distribution, and representation for automatic encoding with no handcrafted encode construction	Provided sufficient data, the method is able to make prediction for any length peptides or allele subtype	70% training set and 30% validation set (Hold-out) and 10-fold cross-validation GPU for faster computation of model SRCC, AUC SRCC = 0.521, 0.521, 0.513 AUC= 0.836, 0.819, 0.818 66.7%
Wang S., 2017, US (Wang et al., 2017)	Analogist	Classification Regression Regularization and optimization	UDNN RNN 2	Prediction of Protein Contact Map	Consists of two major modules, each being a residual neural network	3D models built from contact prediction have Tm score >0.5 for 208 of the 398 membrane proteins	No recognition of predict contact maps from PDB.	Algorithm runs on GPU card. Acc L/k (k= 10, 5, 2, 1) Long-range 47% CCMpred- 21% CASP11–30%
Yeh C., 2017, UK, US (Yeh et al., 2018)	Evolving structures	Optimization GA	GA multithreaded processing	Designed helical repeat proteins (DHRs)	Iterates through mutation, scoring, ranking, and selection	Aims to control the overall shape and size of a protein using existing blocks	First workload imbalance, less efficient work sharing and overheads in scheduling	RMSD value
Simha R., 2015, Canada, Germany, US (Simha et al., 2015)	Bayesian	Classification Probabilistic generative model Bayesian networks	MDLoc BN	Protein multi-location prediction	Each iteration of the learning process obtains a Bayesian network structure of locations using the software package BANJO.	Improvement of MDLoc over preliminary methods with Bayesian network classifiers	MDLoc’s precision values are lower than those of BNCs, MDLoc’s	5-fold cross-validation Presi, Recsi, Acc, F1-scoresi
Yang J., 2015 China, US (Yang et al., 2015)	Analogist	Regression hierarchical order reduction	SVR	Structure prediction of cysteine-rich proteins	Position-specific scoring matrix (PSSM): each oxidized cysteine residue is represented as a vector of 20 elements	Cyscon improved the average accuracy of connectivity pattern prediction	Contact information must be predicted from sequence either by feature-based training or by correlated mutations	10-fold cross-validation and 20-fold cross-validation QC, QP 21.9%
Folkman L., 2014, Australia (Folkman et al., 2014)	Bayesian Constrained optimization	Classification predicted probability of the mutation	SFFS SVM EASE-MM	Model designed for a specific type of mutation	Feature-based multiple models with each model designed for a specific type of mutations	EASE-MM archived balanced results for different types of mutations based on the accessible surface area, secondary structure, or magnitude of stability changes	Using an independent test set of 238 mutations, results were compared in with related work	10-fold cross-validation ROC, AUC, MCC, Q2, Sn, Sp, PVV, NPV AUC = 0.82 MCC = 0.44 Q2 = 74.71 Sn = 73.14 Sp = 75.28 PVV = 52.30 NPV = 88.33
Li Z., 2014, US (Li et al., 2014)	Bayesian	Classification Probability output prediction	SPIN NN	Sequence profile prediction	Sequence Profiles by Integrated Neural network based on fragment-derived Sequence profiles and structure-derived energy profiles	SPIN improves over the fragment-derived profile by 6.7% (from 23.6 to 30.3%) in sequence identity between predicted and raw sequences	Minor improvement in the core of proteins, which have 10% less hydrophilic residues in predicted sequences than raw sequences	10-fold cross-validation MSE, Precision, Recovery rate
Eisenbeis S., 2012, Germany (Eisenbeis et al., 2012)	N/A	N/A	N/A	Enzyme design	No network	No network	No network	—
Qi Y., 2012, US (Qi et al., 2012)	Connectionist	Classification Back propagation in deep layers	DNN	Prediction of local properties in proteins	An amino acid feature extraction layer. A sequential feature extraction layer. A series of classical neural network layers	For the prediction of coiled coil regions, our performance of 97.4% beats the best result (94%) on the same dataset from using the same evaluation setup	The largest improvement is observed for relative solvent accessibility prediction, from 79.2 to 81.0% in the multitask setting	3- and 10-fold cross-validation Acc, precision, recall, F1 80.3%
Ebina T., 2011, Japan (Ebina et al., 2011)	Analogist	Classification Domain linker prediction SVM	DROP SVM RF	Domain predictor	Vector encoding. Random Forest feature selection. SVM parameter optimization. Prediction assessment	Advantage for testing several averaging windows, 600 properties encoded, averaged with five different windows into a 3000-dimensional vector	Computational time required for performing an exhaustive search	5-fold cross-validation AUC, Sn, Precision, NDO, AOS
Yang Y., 2011, US (Yang et al., 2011)	Probability Inference	Regression probabilistic-based matching	SPARKS-X Algorithm	Single-method fold recognition	The model is built by modeller9v7 using the alignment generated by SPARKS-X	SPAKRS-X performs significantly better in recognizing structurally similar proteins (3%) and in building better models (3%)	HHPRED improve 3% over SPARKS-X due to significantly more sophisticated model building techniques	ROC, TPR, FPR
Briesemeister S., 2010, Germany (Briesemeister et al., 2010)	Bayesian	Classification probabilistic approach	NB	Predict protein subcellular localization	Yloc, based on the simple naive Bayes classifier	Small number of features and the simple architecture guarantee interpretable predictions	Returns in confidence estimates that rate predictions are reliable or not	5-fold cross-validation Acc, F1-score, precision, recall
Lin G., 2010, US (Lin et al., 2010)	Analogist	Classification Optimization	SVM SVR	Protein folding kinetic rate and real-value folding rate	SVM classifier to classify folding types based on binary kinetic mechanism (two-state or multi-state), instead of using structural classes of all-α-class, all-β-class and α/β-class	The accuracy of fold rate prediction is improved over previous sequence-based prediction methods	Performance can be further enhanced with additional information	Leave-one-out cross-validation (LOOCV) Classification accuracy surface, Predicted precision
Tian J., 2010, China (Tian et al., 2010)	Analogist	Classification Optimization	RFR SVM RF	Effect on single or multi-site mutation on protein thermostability	Random forest includes bootstrap re-sampling, random feature selection, in-depth decision, tree construction, and out-of-bag error estimates	Overall accuracy of classification and the Pearson correlation coefficient of regression were 79.2% and 0.72	Direct comparison of Prethermut with the other published predictor was not performed as a result of data limitation and differences	10-fold cross-validation Overall accuracy (Q2), MCC, Sn, Sp, Pearson correlation coefficient ® Acc = 79.2% r = 0.72
Zhao F., 2010, US (Zhao et al., 2010)	Bayesian	Classification probabilistic graphical model	CNF SVM	Protein folding	Conformations of a residue in the protein backbone is described as a probabilistic distribution of (θ, τ)	The method generates conformations by restricting the local conformations of a protein	CNF can generate decoys with lower energy but not improve decoy quality	5-, 7-, and 10-fold cross-validation Accuracy (Q3) Q3 = 80.1%
Hong E., 2009, US (Hong et al., 2009)	Symbolist	Classification Branch and bound tree Logical inference	BroMap	Tenth human fibronectin, D44.1 and DI.3 antibodies, Human erythropoietin	BroMAP attempts the reduction of the problem size within each node through DEE and elimination	Lower bounds are exploited in branching and subproblem selection for fast discovery of strong upper bounds	BroMAP is particularly applicable to large protein design problems where DEE/A∗ struggles and can also substitute for DEE/A∗ in general GMEC search	N/A
Özen A., 2009, Turkey (Özen et al., 2009)	Analogists	Classification Regression Constrained optimization	SVM KNN DT SVR	Single-site amino acid substitution	Early Integration. Intermediate Integration. Late Integration	Possible combination including new feature set, new kernel, or a learning method to improve accuracy.	Training any classifier with an unbalanced dataset in favor of negative instances makes it difficult to learn the positive instances	20-fold cross-validation Acc, Error rate, Precision, Recall, FP rate Acc= 0.842, 0.835
Ebrahimpour A., 2008, Malaysia [(Ebrahimpour et al., 2008)	Connectionist	Classification Back and batch back propagation	ANN FFNN IBP BBP QP GA LM	Lipase production Syncephalastrum racemosum, Pseudomonas sp. strain S5 and Pseudomonas aeruginosa	ANN architecture: input layer with six neurons, an output layer with one neuron, and a hidden layer. Transfer functions of hidden and output layers are iteratively determined	Maximum predicted values by ANN (0.47 Uml -1) and RSM (0.476 U–l - 1), whereas R2 and AAD were determined as 0.989 and 0.059% for ANN and 0.95 and 0.078% for RSM, respectively	ANN has the disadvantage of requiring large amounts of training data	RMSE, R2, AAD RMSE<0.0001 R2 = 0.9998
Huang W., 2008, Taiwan (Huang et al., 2008)	Analogist	Clustering Combinatorial optimization	GA SVM KNN	Prediction method for predicting subcellular localization of novel proteins	Preparation of SVM, binary classifiers of LIBSVM. Sequence representation. Inclusion of essential GO terms	Bias-free estimation of the accuracy reduces computational cost	Computational demand is impractical for large datasets	10-fold cross-validation and leave-one-out cross-validation (LOOCV) Accuracy, MCC Acc= 90.6–85.7%
Katzman S., 2008, US (Katzman et al., 2008)	Bayesian	Classification Probabilistic	MUSTER SVM	Local structure prediction	Calculation of output of each unit in each layer. Soft max function to all outputs of a given layer represents valid probability distribution	Accurate predictions of novel alphabets for extending the performance	Smaller windows and number of units, the network has fewer total degrees of freedom	3-fold cross-validation, Qn
Liao J., 2007, US (Liao et al., 2007)	Supervised Learning	Classification Regression	RR Lasso PLSR SVMR LPSVMR LPBoosR MR ORMR	Proteinase K variants	Design of protein variants. Expression of the protein variants. Analysis of protein variant sequences and activities to assess the contribution of each amino acid substitution	Machine learning algorithms make it possible to use more complex and expensive tests to only protein properties	Computational resources are cheap; we instead used the 1000 subsamples of the training sets	Cross-validation
Raveh B., 2007, Israel (Raveh et al., 2007)	Connectionist	Clustering Pattern recognition	K-means Clustering	Existence of α-helices, parallel β-sheets, anti-parallel sheets and loops. Non-conventional hybrid structures	Network motif vector (k means of motif vector). Enriched Interaction graphs	Rediscovery existence of conventional a-helices, parallel b-sheets, anti-parallel sheets and loops, and non-conventional hybrid structures	Limitation to backbone interactions, the degree of each node in the network was bounded from above by two covalent and two possible hydrogen bonds	10-fold cross-validation
Shamim M., 2007, India (Shamim et al., 2007)	Analogist	Classification Regression	SVM	Protein-fold prediction	LIBSVM provides a choice of in-built kernels, such as Linear, Polynomial, Radial basis function (RBF), and Gaussian, we use RBF kernel	Overall accuracy of 65.2% for fold discrimination and individual propensities, which is better than those from the literature	Incrementation of backbone conformation results in the reduction on accuracy prediction	2-fold cross-validation 5-fold cross-validation Accuracy (Q), Sn, Sp Q= 65.2% >70%
Hung C., 2006, Taiwan (Hung et al., 2006)	Symbolist	Regression Genetic algorithm casual tree	DFS HMM GA AGCT	Predict protein functions	AGCT study applies a hybrid methodology based on genetic programming with a causal tree model to predicting protein function	The model is developed to exploit global search capabilities in genetic programming for predicting protein functions of a distantly related protein family that has difficulties in the conserved domain identification	Ratios of comparison between the heuristic signal match and exhaustive sequence alignment are low	Cross-validation
Sidhu A., 2006, UK (Sidhu and Zheng, 2006)	Symbolist	Classification Logical Inference	BBFNN NN DT	Predict signal peptide	BBFNN Characteristics: Mutation matrix for protein sequence encoding. BBFNN is a linear combination of K bio-bases with the bio-basis function	The BBFNN has improved the accuracy by a further 5%. Most cost-effective and efficient way of predicting signal peptides	Size of the positive examples in the dataset reduces prediction accuracy	5-fold cross-validation Accuracy Acc >90%, 97.16% for BBFNN 97.63% for C4.5
Zimmermann O., 2006, Germany, US (Zimmermann and Hansmann, 2006)	Analogist	Classification	SVM C-SVM algorithm implementation	Prediction of dihedral regions	Implementation of the sequence window of length seven and three separate predictions: helix, extended beta, and outliers	Profile-only SVM classifiers show a prediction performance of 80%	The approach is based on sequence profiles only. Models show a tendency to over-predict extended residues and under-predict residues in the helical state	Acc, MCC, Sn, Sp Acc = 93.3%, 93.4% MCC = 0.645, 0.671
Capriotti E., 2005, Italy (Capriotti et al., 2005)	Analogist	Classification	SVM	Protein stability prediction	Prediction of the direction of the protein stability changes upon single-point mutation from the protein tertiary structure	Large extent protein stability can be evaluated with specific interactions in the sequence neighbors captured	Correlation of predicted with expected/experimental values is 0.71 with a standard error of 1.30 kcal/mol and 0.62 with an SE of 1.45 kcal/mol	Cross-validation Accuracy, MCC, Q2 = 0.80, 0.77 MCC = 0.51, 0.42
Rossi A., 2001, Italy (Rossi et al., 2001)	Connectionist	Regression Perceptron algorithm	NN	Barnase and chymotrypsin inhibitor	Two- and three-body energy functions. Partitioning the 20 amino acids into classes (Hydrophobic, Neutral, Charged)	The method is able to identify crucial sites for folding process: for 2ci2 and barnase and shows a very good agreement with experimental results	No improvement on success rate by introducing more sophisticated energy functions. Important features of real proteins are neglected by short-range Hamiltonians	N/A

pre-process

database, pretreatment, and input

process

machine learning paradigm and input, algorithm and development software, three aspects of the neural network used (characteristics, strengths, and limitations) and output.

post-process

input and web server when applied.

**FIGURE 4:**
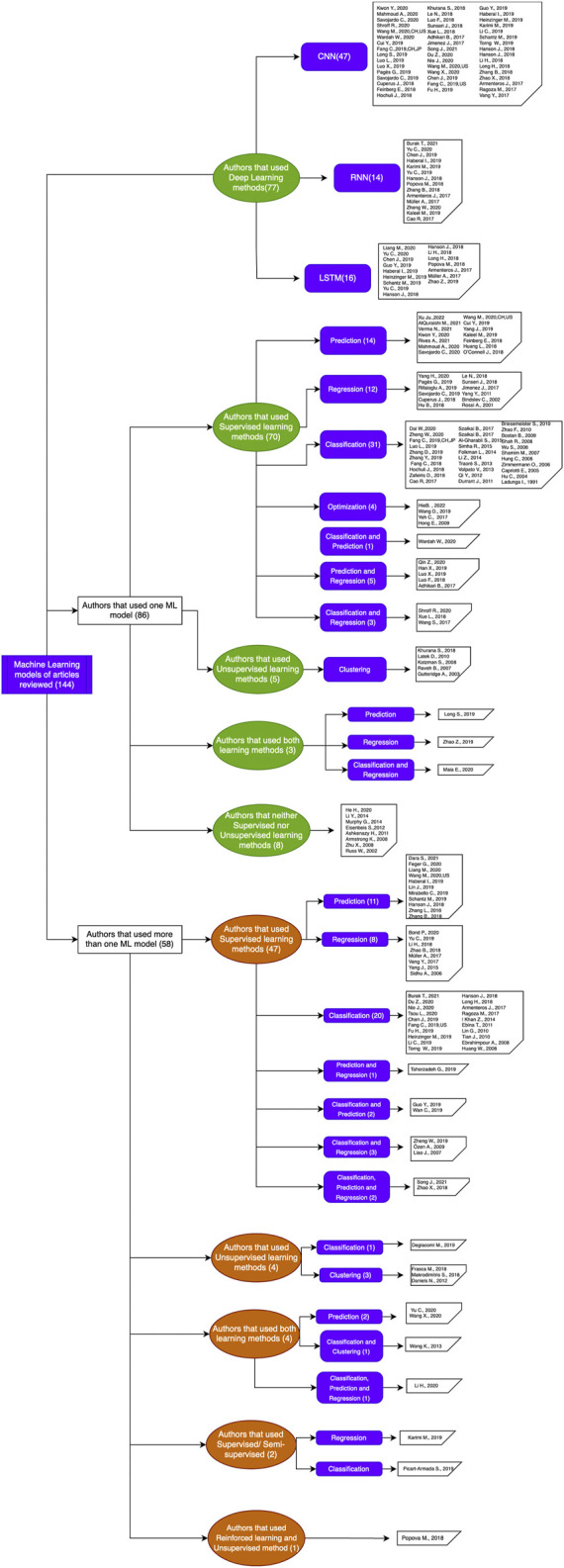
Machine Learning paradigms: superviser learning, unsupervised learning, reinforcement learning.

Most of the research reported in these articles performs a pretreatment over the protein database used, that is, processes of randomization and training, in order to leave the data prepared for the computational process itself, for when the algorithm is to be executed on a software platform and within a particular machine learning paradigm (mostly supervised, unsupervised, and deep learning, as shown in Figure 4). We also reported special characteristics as well as strengths and limitations of the neural networks used. Finally, part of the post-process, when applied, concerns the web server where research results are stored. Moreover, some of these aspects are also registered in Tables 2–6 as well as some others (programming language and software license type).

**TABLE 2:** An overview of the protein and drug design articles with the quality assessment.

First Author/Year of Publication/Country	Database	Initial scaffold (ID)	Designed Protein	ML model	Software/Sever	Programming language/Platform	License	Quality (%)	Machine learning	Protein application
	Protein and drug design									
Hie B., 2022, USA (Hie and Yang, 2022)	N/A	Sequence-to-function machine learning surrogate model t	Protein engineering design	Machine learning optimization	N/A	N/A	N/A	50%	Supervised learning: optimization	Protein design
Dara S., 2021, India (Dara et al., 2021)	ZINC, BindingDB, PUBCHEM, Drugbank, REAL, Genomic Database, Adaptable Clinical Trail Database, DataFoundry, SWISS-PROT, SCoP, dbEST. Genome Information Management System, BIOMOLQUEST, PDB, SWISS-PORT, ENZIME	Target identification, hit discovery, hit to lead, lead optimization	PPI prediction, protein folding, drug repurposing, virtual screening, activity scoring, QSAR, drug design, evaluation of ADME/T properties	AutoEncoder, ANN,CNN, DL, MLP,NB, RF, RNN, CNN, SVM, LR	N/A	N/A	N/A	50%	Supervised learning: prediction	Drug discovery
Feger G., 2020, Czech Republic, France (Feger et al., 2020)	PDB	Peptide amphiphile scaffolds	Amphiphilic peptide scaffold design	SVM, RF	SasFit	C	Open source	60	Supervised Learning: Prediction	Protein design
He H., 2020, China (He et al., 2020)	Multiple databases	Multiple organisms	Review of novel drug discovery techniques	Multiple methods for structure prediction, ligand-binding site, undruggable to drug rabble targets, hidden allosteric site	N/A	N/A	N/A	50	N/A	Drug discovery
Maia E., 2020, Brazil (Maia et al., 2020)	Multiple databases	structure-based virtual screening (SBVS)	Drug development	VSA	N/A	Multiple languages	N/A	60	Supervised Learning: Unsupervised Learning	Drug development design
Qin Z., 2020, US (Qin et al., 2020)	PDB	Phi–psi angle and sequence of natural protein, only of standard amino acids	Protein design of fold alpha-helical structure	MNNN	Tensorflow https://github.com/IBM/mnnn	Python	Open Source	95	Supervised Learning: Prediction Regression	Protein design
Tsou L., 2020, Taiwan (Tsou et al., 2020)	ChEMBL	In-house database of 165,000 compounds	TNBC inhibitors and GPCR classification prediction	DNN, RF	N/A	N/A	N/A	60	Supervised Learning: Classification	Drug design
Wang X., 2020, China (Wang X. et al., 2020)	KIBA, Davis dataset	Kinase protein family	Predict drug-target-binding affinity	CNN, GCN	N/A	N/A	N/A	60	Supervised Learning, Semi-Supervised Learning: Prediction	Drug-target binding-affinity
Yu C., 2020, Taiwan, US (Yu and Buehler, 2020)	PDB	α-helix-rich proteins	*De novo* protein design	RNN, LSTM	TensorFlow, https://github.com/tensorflow/magenta/issues/1438	Python	Open Source	90	Supervised Learning: Unsupervised Learning: Prediction	Protein design
Fang C., 2019, US (Fang et al., 2020)	UniProt	Proteins from datasets BT426 and BT6376 containing at least one beta-turn	Beta-turn prediction	HMM, CNN, DeepDIN	Tensorflow, Keras http://dslsrv8.cs.missouri.edu/∼cf797/MUFoldBetaTurn/download.html	Python	Open Source	90	Supervised Learning: Classification	Protein design
Karimi M., 2019, US (Karimi et al., 2019)	BindingDB, STITCH, Uniref	Various protein classes	Compound–protein affinity prediction	RNN, CNN	https://github.com/ShenLab/DeepAffinity	N/A	N/A	75	Semi-supervised, Unsupervised Learning: Regression	Drug design
Lin J., 2019, China (Lin et al., 2019)	DrugBank	Druggable proteins and non-druggable proteins	Drug target prediction	SVM, GA	https://github.com/QUST-AIBBDRC/GA-Bagging-SVM	Matlab	MathWorks	90	Supervised Learning: Prediction	Drug design
Hu B., 2018, China (Hu et al., 2018)	DDI, SIDER, TWOSIDES, HPRD, Drug Bank, Offsides PubChem	Semantic meta-paths ADR	meta-path-based proximities ADR	SDHINE, Network embedding	TensorFlow, N/A	C, C++, Python	Apache 2.0	65	Supervised Learning: Regression	Drug design
Popova M., 2018, Russia, US (Popova et al., 2018)	PHYSPROP, ChEMBL, KKB	SMILE string	Drug design (*de novo* design)	Stack-RNN, LSTM, ReLeaSE	PyTorch, TensorFlow ReLeaSE https://github.com/isayev/ReLeaSE	Python, CUDA	Open Source	75	Reinforced Learning, Unsupervised Learning: Regression	Drug design
Zafeiris D., 2018, UK (Zafeiris et al., 2018)	GEO, Array Expression	Amyloid beta-precursor protein, microtubule-associated protein tau, apolipoprotein E	Biomarker discovery for Alzheimer’s disease	ANN	N/A	N/A	N/A	50	Supervised Learning: Classification	Enzyme design
Jimenez J., 2017, Spain (Jiménez et al., 2017)	scPDB	PDB ID File or PDB file	Predict protein–ligand-binding sites Drug design	3D-DCNN	Keras, Theano www.playmolecule.org	Python	Open Source	90	Supervised Learning: Regression	Drug design
Müller A., 2017, Switzerland (Müller et al., 2018)	ADAM, APD DADP	Antimicrobial peptide Amino acid sequences	Design of new peptide combinatorial *de novo* peptide design	RNN, LSTM	modlAMP Python package https://github.com/alexarnimueller/LSTM_peptides	Python	Open Source	100	Supervised Learning: Regression	Drug design
Ragoza M., 2017, US (Ragoza et al., 2017)	PDB ChEMBL	Spatial and chemical features of protein–ligand complex	Protein–ligand score for drug discovery	CNN, SGD	Gnina Caffe https://github.com/gnina	C++	Open Source	85	Supervised Learning: Classification	Drug design
Yeh C., 2017, UK, US (Yeh et al., 2018)	JSON database: centers of mass and geometric relationship data	Helical repeat proteins, Center of mass (CoM) using C-α protein sequence	Designed helical repeat proteins (DHRs)	GA multithreaded processing	ELFIN https://github.com/joy13975/elfin	Python, C++, MATLAB	Apache 2.0 open source 3-Clause BSD	90	Supervised Learning: Optimization	Drug design
Folkman L., 2014, Australia (Folkman et al., 2014)	ProTherm	Protein sequence and amino acid substitution	Model designed for a specific type of mutation	EASE-MM, SVM	EASE-MM LISVM http://www.ict.griffigr.edu.au/bioinf/ease	Python, Linux	Open Source	75	Supervised Learning: Classification	Model design
Khan Z., 2014, Pakistan (Khan et al., 2015)	BRENDA	Amino Acid sequence and alkaline enzyme	Enzyme catalysis	DT, KNN, MLP, PNN, SVM	MATLAB Bioweka Weka	Java	Open Source MathWorks	50	Supervised Learning: Classification	Drug design
Li Y., 2014, US (Li and Cirino, 2014)	PDB	E. coli	Designs of improved enzymes and enzymes with new functions and activities	Computational design and scaffolding and compartmentalization	N/A	N/A	N/A	50	N/A	Drug design
Murphy G., 2014, US (Murphy et al., 2015)	DND_4HB protein	DND_4HB protein	Design an up-down four-helix bundle	Computational folding	N/A	N/A	N/A	50	N/A	Drug design
Traoré S., 2013, France (Traoré et al., 2013)	PDB	3D protein structure	Structure-based computational protein design framework	CFN	CPD http://genoweb.toulouse.inra.fr/tschiex/CPD	Perl	Open source	65	Supervised Learning: Classification	Protein design
Volpato V., 2013, Ireland (Volpato et al., 2013)	ENZYME UniProt	Oxidoreductase, transferase, hydrolase, lyase, isomerase, and ligase	Acid-residue frequency derived from multiple sequence alignments extracted from uniref90	N-to-1 Neural Network	N/A	N/A	N/A	65	Supervised Learning: Classification	Drug design
Daniels N., 2012, US (Daniels et al., 2012)	SCOP	Protein sequence, 207 beta structural SCOP super families	Detection for beta-structural proteins into the twilight zone, make over a 100-new-fold prediction genome of T. maritima	HMM, MRF	SMURFLite http://smurf.cs.tufts.edu/smurflite/	N/A	Open Source	65	Unsupervised Learning: Clustering	Drug design
Eisenbeis S., 2012, Germany (Eisenbeis et al., 2012)	PDB	(βα)8-barrel and the flavodoxin-like fold, CheY, HisF	Enzyme design	Rational recombination	http://pubs.acs.org Modeller, Rosetta	Python	IBM, Academic nonprofit freeware	75	N/A	Drug design
Ebina T., 2011, Japan (Ebina et al., 2011)	DS-All dataset	Protein sequence	Domain predictor	DROP, SVM, RF	DROP http://web.tuat.ac.jp/∼domserv/DROP.html	Bash script	Open source	75	Supervised Learning: Classification	Drug design
Bostan B., 2009, US (Bostan et al., 2009)	KEGG	Given a species proteome	Predict homologous signaling pathway	PSP	N/A	N/A	N/A	50	Supervised Learning: Classification	Model design
Hong E., 2009, US (Hong et al., 2009)	Standard rotamer library Expanded rotamer library	Fn3: Derived from protein Fn3, 10th human fibronectin-type III domain	Tenth human fibronectin, D44.1 and DI.3 antibodies, Human erythropoietin	BroMAP	BroMAP	C++, Linux	Open Source	100	Supervised Learning: Optimization	Drug design
Özen A., 2009, Turkey (Özen et al., 2009)	ProTherm	Structure-based features: amino acid substitution likelihood equilibrium fluctuations α, Cβ, packing density	Single-site amino acid substitution	SVM, KNN, DT, SVR	MOSEK http://www.prc.boun.edu.tr/appserv/prc/mlsta	N/A	Open Source	85	Supervised Learning: Classification Regression	Model design
Ebrahimpour A., 2008, Malaysia (Ebrahimpour et al., 2008)	GenBank	Geobacillus sp. Strain	Lipase production Syncephalastrum racemosum, Pseudomonas sp. Strain S5 and Pseudomonas aeruginosa	ANN, FFNN, IBP, BBP, QP, GA, LM	CPC-X Software N/A	Java	Neural Power version 2.5	75	Supervised Learning: Classification	Protein design
Zhu X., 2008, China (Zhu and Lai, 2009)	PDB	223 scaffold proteins	Pocket residues of ribose-binding protein (2dri), tyrosyl-t/RNA synthetase (4ts1), and tryptophan synthase (1a50). No metal ion-binding sites	Vector matching	N/A	N/A	N/A	65	N/A	Drug design
Liao J., 2007, US (Liao et al., 2007)	GenBank	Proteinase K-catalyzed hydrolysis of the tetrapeptide N-Succinyl-Ala-Ala-Pro-Leu p-nitroanilide	Proteinase K variants	RR, Lasso, PLSR, SVMR, LPSVMR, LPBoosR, MR, ORMR	N/A	Matlab	MathWorks	75	Supervised Learning: Classification Regression	Protein design
Raveh B., 2007, Israel (Raveh et al., 2007)	PDB	TIM-barrel fold 1YPI. Whole β-sheet global structures	Existence of α-helices, parallel β-sheets, anti-parallel sheets and loops. Non-conventional hybrid structures	K-means clustering	Matlab	Matlab	MathWorks	75	Unsupervised Learning: Clustering	Protein design
Zimmermann O., 2006, Germany (Zimmermann and Hansmann, 2006)	PDB	Protein sequence	Prediction of dihedral regions	C-SVM	LIBSVM-library DHPRED http://www.fz- juelich.de/nic/cbb	C, Python, Linux, Windows	Open source	80	Supervised Learning: Classification	Protein design
Russ W., 2002, US (Russ and Ranganathan, 2002)	N/A	SH3 domain GroEL minichaperone WW domain prototype	Thermostable consensus phytase, 84.5 kDa protein	Knowledge-base potential functions	N/A	N/A	N/A	65	N/A	Protein design
Rossi A., 2001, Italy (Rossi et al., 2001)	PDB, HSSP	2ci2 Barnase	Barnase and chymotrypsin inhibitor	Perceptron	N/A	N/A	N/A	90	Supervised Learning: Regression	Drug design

3D-CNN, Three-dimensional convolutional neural network; ANN, Artificial neural network; BBP, Back Back propagation; BroMap, Branch and bound map estimation; CFN, Cost function network; CNN, Convolutional neural network; DeepDIN, Deep dense inception network; DT, Decision tree; DROP, Domain linker prediction using optimal feature; EASE-MM, Evolutionary Amino acid, and Structural Encodings with Multiple Models; FFNN, Feed forward neural network; GA, Genetic algorithms; GCN, Graph convolutional network; HMM, Hidden Markov model; IBP, Incremental back propagation; KNN, k-nearest neighbor; Lasso, Least absolute shrinkage and selection operator; LM, Levenberg–Marquardt; LPBoostR, Linear programming boosting regression; LPSVMR, Linear programming support vector machine regression; LSTM, Long short-term memory; MLP, Multilayer perceptron; MR, Matching loss regression; MRF, Markov random forest; MNNN, Multi-scale neighborhood-based neural network; ORMR, One-norm regularization matching-loss regression; PLSR, Partial least-squares regression; PNN, Probabilistic neural network; PSP, Predict Signal Pathway; QP, quick prob; ReLeaSE, Reinforcement Learning for Structural Evolution; RF, Random forest; RNN, Recurrent neural network; RR, Ridge regression; SDHINE, Meta path-based heterogeneous information embedding approach; SFFS, Sequential forward floating selection; SGD, Stochastic gradient descent; SVM, Support vector machine; SVMR, Support vector machine regression; SVR, Support vector regression; VSA, Virtual screening algorithms.

**TABLE 3:** An overview of the protein function prediction, function prediction, and novel function articles with the quality assessment.

First Author/Year of Publication/Country	Database			Initial scaffold (ID)	Designed Protein	ML model	Software/Sever	Programming language/Platform	License	Quality (%)	Machine learning	Protein application
		Protein function prediction										
Verma N., 2021, US (Verma et al., 2021)	DrugBank matador PDB			Human, *C. Elegans*	Protein–ligand interactions	DNN	GitHub (https://github.com/ekraka/SSnet)	Python	Open source	75	Supervised learning: Prediction	Protein–ligand interaction prediction
Du Z., 2020, China, Russia, US (Du et al., 2020)	CAFA3, SwissProt			Human, *C. Elegans*	Automated function prediction	NLP, CNN	Keras, TensorFlow	Python	Open Source	70	Supervised Learning: Classification	Protein function prediction
Liang M., 2020, China (Liang and Nie, 2020)	PDB			Relative angle of (C – Cα – C) principal plane	Enzymatic function prediction	RN, LSTM	TensorFlow	Python	Open Source	90	Supervised Learning: Prediction	Protein function prediction, Function ID
Rifaioglu A., 2019, Turkey, UK (Rifaioglu et al., 2019)	UniProtKB/Swiss-Prot			N/A	GO term prediction	DNN	Tensorflow, https://github.com/cansyl/DEEPred	Python	Open Source	70	Supervised Learning: Regression	Protein function prediction
Torng W., 2019, US (Torng and Altman, 2019)	PROSITE NOS dataset			Protein structure as 3D images	Protein functional site detection	DL, 3D-CNN, SVM	N/A https://simtk.org/projects/fscnn	Python	N/A	75	Supervised Learning: Classification	Protein function prediction
Wan C., 2019, UK (Wan et al., 2019)	UniProtKB/Swiss-Prot			Human proteins	Function prediction	DMNN, SVM	Keras, https://github.com/psipred/STRING2GO	Python	Open Source	80	Supervised Learning: Prediction Classification	Protein function prediction
Feinberg E., 2018, China, US (Feinberg et al., 2018)	PDB Bind 2007			Scaffold split for grouping ligands in common frameworks	Molecular Property Prediction	GCNN	PyTorch, NumPy and SciPy	Python	Open Source	100	Supervised Learning: Prediction	Protein function prediction
Frasca M., 2018, Italy (Frasca et al., 2018)	STRING GO			Organisms: Homo sapiens (human) S. cerevisiae (yeast) Mus musculus (mouse)	AFP (Automated Protein Function Prediction)	COSNet, ParCOSNet, HNN	COSNet, ParCOSNet	C, C++, R, CUDA	Open Source	75	Unsupervised Learning: Clustering	Protein function prediction
Khurana S., 2018, Qatar, US (Khurana et al., 2018)	pepcDB database			k-mer structure and additional sequence and structural features extracted from the protein sequence	Solubility prediction	CNN, DL, FFNN	PROSO II https://zenodo.org/record/1162886#.XSP26ffPzOQ DeepSol: https://github.com/sameerkhurana10/DSOL_rv0.2	Python, Linux	Open source	95	Unsupervised Learning: Clustering	Protein function prediction
Li H., 2018, China (Li et al., 2018)	HPRD DIP HIPPIE			Primary sequence Escherichia coli, Drosophila, Caenorhabditis elegans, Pan’s PPI datasets	Prediction of protein interactions	DNN, CNN, LSTM	Keras, Theano, TensorFlow, N/A	Python	Open Source	85	Supervised Learning: Regression	Protein function prediction
Long H., 2018, China, US (Long et al., 2018)	UniProt			PseAAC Hydroxyproline and hydroxylysine	Predicting hydroxylation sites	CNN, LSTM	MXNet, N/A	R	Apache 2.0	85	Supervised Learning: Classification	Protein function prediction
Makrodimitris S., 2018, Netherlands (Makrodimitris et al., 2019)	Arabidopsis thaliana proteins			Arabidopsis thaliana protein	Protein function prediction	KNN, LSDR	SciPy https://github. Com/stamakro/SSP-LSDR.	Python, MATLAB Bioinformatics toolbox	Open source, Mathworks	80	Unsupervised Learning: Clustering	Protein function prediction
Zhang L., 2018, China (Zhang L. et al., 2018)	UniProt, DIP			S. cerevisiae, H. sapiens, and M. musculus	Predicting Protein–Protein interactions	DNN, Adam Algorithm	TensorFlow	Python	Open Source	100	Supervised Learning: Prediction	Protein function prediction
Adhikari B., 2017, US (Adhikari et al., 2018)	DNCON Dataset			N/A	Contact map protein prediction	CNN	TensorFlow, Keras http://sysbio.rnet.missouri.edu/dncon2/	Python	Open Source	65	Supervised Learning: Regression Predection	Protein residue–residue contacts
Cao R, 2017, US (Cao et al., 2017)	UniProt			Protein sequence	Protein function prediction	RNN	ProLanGO Model N/A	N/A	N/A	50	Supervised Learning: Classification	Protein function prediction
Al-Gharabli S., 2015, Jordan (Al-Gharabli et al., 2015)	PDB			Amino acid sequence hydrophobicity	Prediction of dihedral angles physiochemical properties, enzyme loops	ANN	N/A	N/A	N/A	50	Supervised Learning: Classification	Protein function prediction
Qi Y., 2012, US (Qi et al., 2012)	Standard benchmark, CB513 DSSP			PSI-BLAST amino acid embedding	Prediction of the local properties in proteins	DNN	Torch5	C	Open Source	100	Supervised Learning: Classification	Protein function prediction
Yang Y., 2011, US (Yang et al., 2011)	SPINE			Protein sequence	Single-method fold recognition	SPARKS-X Algorithm	SPARKS-X https://sparks-lab.org/server/sparks-x/	Shell script	Open Source	75	Supervised Learning: Regression	Protein function prediction
Latek D., 2010, Poland (Latek and Kolinski, 2011)	10 globular proteins, 216 residues, and S100A1 protein			10 globular proteins and S100A1 protein	Predicted Nuclear Overhauser Effect signals on the basis of low-energy structures from CABS-NMR	CABS, MC	CABS- NMR toolkit http://biocomp.chem.uw.edu.pl/services.php	N/A	N/A	70	Unsupervised Learning: Clustering	Protein function prediction
Tian J., 2010, China, US (Tian et al., 2010)	ProTherm PDB			3D structures	Effect on single- or multi-site mutation on protein thermostability	RFR, RF, SVM	Prethermut http://www.mobioinfor.cn/prethermut/	R, Perl, Linux	Open Source	75	Supervised Learning: Classification	Protein function prediction
Wu S., 2008, US (Wu and Zhang, 2008)	PDB			PDB protein sequence	Protein contact predictor	MUSTER	MUSTER http://zhang.bioinformatics.ku.edu/MUSTER	N/A	N/A	50	Supervised Learning: Classification	Protein function prediction
Hung C., 2006, Taiwan (Hung et al., 2006)	NCBI			Nucleocapsid (nsp1) of a coronavirus family	Predict protein functions	AGCT	N/A	N/A	N/A	75	Supervised Learning: Classification	Protein function prediction
Sidhu A., 2006, UK (Sidhu and Zheng, 2006)	Swiss-Prot			Signal peptides and non-secretory proteins from Human, E. coli, prokaryotic	Predict signal peptide	BBFNN, DT	N/A	N/A	N/A	75	Supervised Learning: Regression	Protein function prediction
Capriotti E., 2005, Italy (Capriotti et al., 2005)	ProTherm			Protein tertiary structure	Protein stability prediction	SVM	I-Mutant2.0 http://gpcr.biocomp.unibo.it/cgi/predictors/I-Mutant2.0/I-Mutant2.0.cgi	Python	Open Source	75	Supervised Learning: Classification	Protein function prediction
Hu C., 2004, US (Hu et al., 2004)	WhatIF database UniProt			3D coarse-grained structure from protein sequences	Optimal non-linear scoring	SVM non-linear Gaussian kernel functions	N/A	N/A	N/A	65	Supervised Learning: Classification	Protein function prediction
Gutteridge A., 2003, UK (Gutteridge et al., 2003)	PDB			Amino acid sequence of quinolate phosphoribosyl transferase	Predict active site	FFNN	N/A	N/A	N/A	50	Unsupervised Learning: Clustering	Protein function prediction
		Function Prediction and Novel Function										
Nie J., Singapore 2020 (Sua et al., 2020)	UniProt			acetyl-lysine (S1), “crotonyl-lysine” (S2), “methyl-lysine” (S3), or “succinyl-lysine” (S4)	Identification of Lysine PTM sites	RF, SVM, MNB, LR, ME, KNN, CNN, MLP	Tensorflow, https://github.com/khanhlee/lysineSGT	Python	N/A	100	Supervised Learning: Classification	Function ID
Savojardo C.., 2020, Italy (Savojardo et al., 2020a)	UniProtKB GOA, DeepMitoDB			Human, mouse, fly, yeast, and Arabidopsis thaliana	protein sub-mitochondrial localization	DeepMito, 1D-CNN	N/A	N/A	N/A	75	Supervised learning: Prediction	Function ID
Fang C., 2019, China, Japan (Fang et al., 2019)	PDB			MoRF-containing membrane protein chains	Molecular recognition features MoRFs prediction	DCNN	N/A	N/A	N/A	75	Supervised Learning: Classification	Function ID and Fold ID
Zhang Y., 2019, China (Zhang et al., 2019)	PDB			PDNA-543, PDNA-224 and PDNA-316	Identification of DNA–protein-binding site	ADASYN	Theano	Python	Open Source	85	Supervised Learning: Classification	Function ID and Fold ID
Hanson J., 2018, Australia, China (Hanson et al., 2019)	PISCES CASP12 PDB			5N5EA 6FI2A 6FQ3A	Sequence-based prediction of one-dimensional structural properties of proteins	CNN, 2D-BRLSTM	N/A	N/A	N/A	80	Supervised Learning: Classification	Function ID
Shah R., 2008, US (Shah et al., 2008)	D Dataset			Protein sequence	Homology detection	SVM	SVM-HUSTLE http://www.sysbio.org/sysbio/networkbio/svm_hustl	N/A	N/A	70	Supervised Learning: Classification	Function ID and Fold ID

1D-CNN, one-dimensional convolutional neural network; 2D-BRLSTM, two-dimensional bidirectional recurrent long short-term memory; 3D-CNN, three-dimensional convolutional neural network; ADASYN, Adaptive Synthetic Sampling; ANN, Artificial neural network; AGCT, Alignment genetic causal tree; BBFNN, Biobasis function neural network; CABS, C-alpha-beta side; CNN, Convolutional neural network; COSNet, Cost-sensitive neural network; DCNN, Deep Convolutional neural network; DMNN, Deep mahout neural network; DFS, Depth first search; DL, Deep learning; DNN, Deep neural network; DTNN, Deep tensor neural network; FFNN, Feed forward neural network; GA, Genetic algorithms; HDL, Hybrid Deep learning; HMM, Hidden Markov model; HNN, Hopfield neural network; KNN, k-nearest neighbor; LR, Logistic regression; LSDR, Label-Space dimensionality reduction; LSTM, Long short-term memory; MC, Monte Carlo; ME, Max Entropy; MLP, Multilayer; MNB, Multinomial Naïve Bayes; MNPP, Message passing neural network; NLP, Natural language processing; NN, Neural network; ParCOSNet, Parallel COSNet; RF, Random forest; RN, Relational network; RNN, Recurrent neural network; SPARK-X, Probabilistic-based matching; SVM, Support vector machine.

**TABLE 4:** An overview of the fold id, physicochemical properties, and protein classification articles with the quality assessment.

First Author/Year of Publication/Country	Database	Initial scaffold (ID)	Designed Protein	ML model	Software/Sever	Programming language/Platform	License	Quality (%)	Machine learning	Protein application
	Fold ID and physicochemical properties									
Rives A., 2020, UK, USA (Rives et al., 2021)	SCOPe	Protein data in the form of unlabeled amino acid sequences. Small vocabulary of 20 canonical elements	Predicted model contains information about biological properties in its representations	Deep contextual language model	https://github.com/facebookresearch/esm	Python	Open source	70	Supervised learning; prediction	Physicochemical and biological properties
Li H., 2020, France, Hong Kong (Hongjian et al., 2021)	PDB, PubChem, ZINC, ChEMB,L BindingDB, HTS	Chemical Estrogen receptor α (Erα) Anaplastic lymphoma kinase Neuraminidase (NA) Reducing the level of Dmiro protein in flies Acetylcholinesterase (AchE)	Protein–ligand complex	RF, BRT, kNN, NN, SVM, GBDT, multi-task DNN XGBoost	Descriptor data bank ODDT BINANA RF-Score-v1 RF-Score-v3 MIEC-SVM	Python	Open Source	100	Supervised Learning; Unsupervised Learning; Prediction Classification Regression	Physicochemical properties
Shroff R., 2020, US (Shroff et al., 2020)	PDB	N/A	amino acid association guide mutation	3D CNN	Theano www. Mutcompute.com	Python	Open Source	70	Supervised Learning: Class Prediction	Microenvironment mutation identification
Wang M., 2020, China, US (Wang M. et al., 2020b)	UniProt	E. coli, M. musculus, H. sapiens	Protein malonylation site prediction	DL-CNN	Keras, https://github.com/QUST-AIBBDRC/DeepMal/	Python, Matlab	Open Source	80	Supervised Learning: Classification	Malonylation site prediction
Chen J., 2019, China (Chen et al., 2019)	Datasets A(CPLM),B,C	Proteins and reducing sugars	Glycation product prediction	RNN, CNN	N/A	N/A	N/A	60	Supervised Learning: Classification	Glycation site predictor
Han X., 2019, Singapore, US (Han et al., 2019)	eSol	Cell-free protein expression from E. coli	Protein solubility	GAN	N/A	N/A	N/A	60	Supervised Learning: Regression Prediction	Protein solubility prediction
Heinzinger M., 2019, Germany (Heinzinger et al., 2019)	UniProt, PDB	TS115 CB513 CASP12	Protein sequence representation	NLP, ELMo	Pytorch, https://embed.protein.properties/	Python	Open Source	80	Supervised Learning: Classification	Fold ID
Kaleel M., 2019, Ireland (Kaleel et al., 2019)	PDB	Amino acids are subcellular into four classes involving RSA	Prediction of relative solvent accessibility	BRNN	http://distilldeep.ucd.ie/paleale/	Python	Open Source	90	Supervised Learning: Prediction	Protein relative solvent accessibility prediction
Li C., 2019, China (Li and Liu, 2020)	LE dataset from SCOP	Multiple superfamilies	Detect the structural motifs related with the protein folds	MotifCNN and MotifDCNN SVM CNN	TensorFlow	Python	Open source	100	Supervised Learning: Classification	Fold ID
Luo L., 2019, China (Luo L. et al., 2019)	BioCreative II, BioCreative III, BioCreative II.5	PPI protein articles	Protein–protein interaction	KeSACNN	Keras	Python	Open Source	50	Supervised Learning: Classification	Physicochemical properties
Taherzadeh T., 2019, Australia, US (Taherzadeh et al., 2019)	Uniprot, dbPTM, Uniprep, UnicarKB, GlycoProtDB	Glycoprotein	N- and O-linked glycosylation	DNN SVM	TensorFlow, https://sparks-lab.org/server/sprint-gly/	Python	Open Source	80	Supervised Learning: Regression Prediction	Glycosylation site identification
Zhang D., 2019, US (Zhang and Kabuka, 2019)	DIP, HPRD, UniProt	D. melanogaster, S. cerevisiae, E. coli, C. elegans, H. sapiens, H. pylori, M. musculus, R. norvegicus	Protein–protein interactions and protein family prediction	Multimodal DNN	N/A	N/A	N/A	75	Supervised Learning: Classification	Physicochemical properties
Cuperus J., 2018, US (Cuperus et al., 2017)	5′ UTR library of 50-nt-long random sequences	Yeast Saccharomyces cerevisiae	Predict protein expression	CNN	Keras, Theano, https://github.com/Seeliglab/2017---Deep-learning-yeast-UTRs	Python	Open Source	85	Supervised Learning: Regression	Fold ID
Hochuli J., 2018, US (Hochuli et al., 2018)	PDB	Ligands SMILE Protein FASTA	Identify protein–ligand scoring	CNN	Gnina, Caffe Github.com/gnina	C++, Python	Open source	50	Supervised Learning: Classification	Protein Scoring
Luo F., 2018, China (Luo F. et al., 2019)	Phospho.ELM, PhosphositePlus, HPRD, dbPTM, SysPTM	Kinase protein family	Protein phosphorylation	CNN	https://github.com/USTCHIlab/DeepPhos	N/A	N/A	60	Supervised Learning: Regression Prediction	Phosphorylation site predictor
Zhao X., 2018, China (Zhao et al., 2018)	PLMD	Lysine	Lysine acetylation sites	CNN DNN	Keras, Theano, https://github.com/jiagenlee/DeepAce	Python	Open Source	80	Supervised Learning: Regression Classification Prediction	Acetylation site prediction
Zhao F., 2010, US (Zhao et al., 2010)	CASP	(PSSM) Position-specific scoring matrix generated by PSI-BLAST	Protein folding	CNF	CNF	N/A	N/A	80	Supervised Learning: Classification	Fold ID
Armstrong K., 2008, US (Armstrong and Tidor, 2008)	PDB	Protein sequence	Protein engineering space of foldable sequences	Computational mapping	N/A	C++	Open source	50	N/A	Fold ID
Shamim M., 2007, India (Shamim et al., 2007)	D-B dataset Ext. D-B dataset	Structural information of amino acid residue and amino acid residue pairs	Protein fold prediction	SVM	LIBSVM-library	C++, Java, Python Windows, Linux	Open source	80	Supervised Learning: Classification	Fold ID
	Protein Classification									
Burak T., 2021, Turkey (Alakuş and Türkoğlu, 2021)	UniProt	Protein sequence from 60 different families	Protein family classification/identification	FIBHASH	N/A	N/A	N/A	70	Supervised Learning: Classification	Protein classification
Zhao Z., 2019, China (Zhao and Gong, 2019)	Monomers and dimers from the author	Monomers and dimers from the author	Protein–protein interaction	LSTM	N/A	N/A	N/A	60	Supervised Learning: Unsupervised Learning: Regression	Interface residue pair prediction
Huang L., 2018, US (Huang et al., 2018)	DIP, HPRD	PPI network graph	Protein–protein interaction	ENN-RL	TensorFlow, https://www.eecis.udel.edu/∼lliao/enn/	Python	Open Source	75	Supervised Learning: Prediction	Protein–protein interaction
Le N., 2018, Taiwan (Le et al., 2018)	UniProt GO	Rab GGT activity Rab GDI activity Rab GTPase binding Rab GEF activity	Classify Rab protein molecules	2D-CNN	Keras, Theano DeepRab; http://bio216.bioinfo.yzu.edu.tw/deeprab/	Python	Open Source	90	Supervised Learning: Regression	Protein Classification
Xue L., 2018, China, US (Xue et al., 2019)	Swiss-Prot, TrEMBL	Secretory protein	Protein sequence into T3Ses or non-T3Ses	DCNN	Keras, https://github.com/lje00006/DeepT3	Python	Open Source	60	Supervised Learning: Regression Classification	Protein classification
Zhao B., 2018, US (Zhao and Xue, 2018)	DisProt PDB	Intrinsically disordered proteins (IDPs), intrinsically disordered regions (IDRs), and intrinsically disordered amino acids (IDAAs)	N/A	ANN, DT	DisEMBL, IUPred, VSL2, Dbann, and Espritz	N/A	N/A	50	Supervised Learning: Regression	Intrinsically disordered protein prediction
Szalkai B., 2017, Hungary(Szalkai and Grolmusz, 2018a)	Swiss-Prot, UniProt, GO	Thyroid hormone, phenol-containing compound, cellular modified amino acid, protein kinase superfamily	protein classification by amino acid sequence	ANN	TensorFlow	Python	Open Source	90	Supervised Learning: Classification	Protein Classification
Szalkai B., 2017, Hungary (Szalkai and Grolmusz, 2018b)	UniProt GO	Classes.tre	Hierarchical Biological Sequence Classification	DNN	SECLAF, TensorFlow https://pitgroup.org/seclaf/	Python	Open Source	85	Supervised Learning: Classification	Protein Classification

3D-CNN, three-dimensional convolutional neural network; ANN, Artificial neural network; BLSTM, Bidirectional long short-term memory; BRNN, Bidirectional recurrent neural network; BRT, Booster regression tree; CNF, Conditional neural filed; DNN, Deep neural network; DT, Decision Tree; ELMO, Embeddings from language models; ENN-RL, Evolution neural network-based Regularized Laplacian kernel; FIBHASH, Fibonacci numbers and hashing table; GAN, Generative adversarial network; GBDT, Gradient boosted decision tree; GR, Genetic recombination; KNN, k-nearest neighbor; KeSCANN, Knowledge-enriched Self-Attention convolutional neural network; LSTM, Long short-term memory; MotifCNN, Motif convolutional neural network; Motif DNN, Motif deep neural network; Multimodal DNN, Multimodal deep neural network; NLP, Natural language processing; NN, Neural network; RF, Random forest; RNN, Recurrent neural network; SPARK-X, Probabilistic-based matching; SVM, Support vector machine.

**TABLE 5:** An overview of the protein structure prediction articles with the quality assessment.

First Author/Year of Publication/Country	Database	Initial scaffold (ID)	Designed Protein	ML model	Software/Sever	Programming language/Platform	License	Quality (%)	Machine learning	Protein application
	Protein Structure Prediction									
Xu J., 2022, USA (Xu et al., 2021)	CASP13, PDB, PISCES, CATH	Discrete probability over distance for three backbone atom pair and inter-residue orientation	Structure prediction	Convolutional residual neural network	https://github.com/j3xugit/RaptorX-3DModeling/	python	Open source	70	Supervised Learning; Prediction	Protein structure prediction
ALQuraishi M., 2021, USA (AlQuraishi, 2021)	PDB, CASP14	Primary protein sequence	Structure prediction	Markov random field, Attention networks	N/A	N/A	N/A	50%	Supervised Learning: Prediction	Protein structure prediction
Bond P., 2020, UK (Bond et al., 2020)	PDB	Only residues with side chains longer than beta-carbon	Predicting the correctness of protein residues	NN, MLP	CCP4	C++, Python	Open Source	60	Supervised Learning: Regression	Protein structure prediction
Wardah W., 2020, Australia, Fiji, Japan, US (Wardah et al., 2020)	BioLiP	Positive (binding) or negative (non-binding), protein sequence classification	Predicting Protein-peptide-binding sites	CNN	PyTorch, https://github.com/WafaaWardah/Visual	Python	Open Source	100	Supervised Learning: Prediction Classification	Protein structure prediction
Yang J., 2019, China, USA (Yang J. et al., 2020)	CASP13, Uniclust30	Representation of the rigid-body transform from one residue to another; angles and distances	Predicted inter-residue orientations	Deep residual convolutional neural network	https://yanglab.nankai.edu.cn/trRosetta/	Python	Open source	70	Supervised Learning; Prediction	Protein structure prediction
Degiacomi M., 2019, UK (Degiacomi, 2019)	PDB	Malate dehydrogenase (1MLD), αB crystallin (2WJ7) Phospholipase A2 (1POA), Envelope glycoprotein (1SVB), MurD, closed (3UAG), MurD, open (1E0D), MurD, closed + open (3UAG,1E0D), HIV-1 (1E6J)	Enhancement of molecular conformational space generator	Molecular dynamics, RF, auto encoder	Keras, Tensorflow	Python	Open Source	80	Unsupervised Learning: Classification	Protein conformational space
Guo Y., 2019, US (Guo et al., 2019)	CB513, CASP10, CASP11	Protein sequences	Protein secondary structure	ACNN, BLSTM	Keras, Tensorflow, https://github.com/GYBTA/DALSTM/	Python	Open Source	80	Supervised Learning: Prediction Classification	Protein secondary structure prediction
Long S., 2019, China (Long and Tian, 2019)	Jpred dataset cullpdb dataset UniRef90 UniProt	Multiple superfamilies	Protein secondary structure prediction	CNN	TensorFlow N/A	Python	Open Source	60	Supervised Learning; Unsupervised Learning; Prediction	Protein structure prediction
Mirabello C., 2019, Sweden (Mirabello and Wallner, 2019)	PDB	N/A	Method prediction	NLP, DNN	Keras, TensorFlow https://bitbucket.org/clami66/rawmsa	Python	Open Source	70	Supervised Learning: Prediction	Protein structure prediction
Pagès G., 2019, France (Pagès et al., 2019)	CASP	Model QA	Protein model quality assessment	3D CNN	TensorFlow, Ornate https://team.inria.fr/nanod/software/Ornate/	C++, Python	Open Source	85	Supervised Learning: Regression	Model protein prediction
Schantz M., 2019, Argentina, Denmark, Malaysia (Klausen et al., 2019)	PDB, PISCES	Crystal structures	Prediction of protein structural features	CNN, LSTM	Keras	Python	Open source	100	Supervised Learning: Prediction	Protein structure prediction
Wang D., 2019, China (Wang D. et al., 2020)	CASP11, 12	Caspase 14	Protein structure refinement	Multi-objective PSO	AIR 2.0 www.csbio.sjtu.edu.cn/bioinf/AIR/	Python	Open Source	95	Supervised Learning: Optimization	Protein structure prediction
Yu C., 2019, US (Yu et al., 2019)	PDB	194l (lysozyme), 107m (myoglobin), 6cgz (β-barrel), a silk protein, amyloid protein, and others	Generation of audible sound from amino acid sequence for application on designer materials	RNN, LSTM	Magenta TensorFlow, Melody RNN	Java, Python	Open Source	100	Supervised Learning: Regression	Protein sequence prediction
Zheng W., 2019, US (Zheng et al., 2019)	CASP13	Query sequence profiles	Automated structure prediction pipeline	ZhangServer and QUARK pipelines	Zhang and Quark server	N/A	Open Source	85	Supervised Learning: Classification Regression	Protein structure prediction
Fang C., 2018, US (Fang et al., 2018)	PDB JPRED CASP CB513	Different super-families, CASP10, 11, 12	Protein secondary structure prediction	Deep3I network	MUFOLD-SS TensorFlow and Keras	Python	Open Source	80	Supervised Learning: Classification	Protein structure prediction
O’Connell J., 2018, Australia, China, US (O’Connell et al., 2018)	SPIN dataset	N/A	Sequence profile compatible	DNN	http://sparks-lab.org. SPIN	N/A	Open Source	65	Supervised Learning: Prediction	Protein sequence prediction
Sunseri J., 2018, US (Sunseri et al., 2019)	D3R Grand challenge 3 Grand challenge 3	Input ligand SMILES protein FASTA CSAR	Cathepsin S model ligand protein	CNN	Gnina, Caffe, https://github.com/gnina	C++, Python	Open Source	100	Supervised Learning: Regression	Protein model prediction
Zhang B., 2018, China (Zhang B. et al., 2018)	PDB, PISCES, TR5534 Dataset	CASP10, 11, 12 and 13	Prediction of performance of protein	CNN, RNN, BRNN	Keras	Python	Open Source	100	Supervised Learning, Prediction	Protein structure prediction
Armenteros J., 2017, Denmark (Almagro Armenteros et al., 2017)	UniProt	Protein sequence, Sequence information	Predict protein subcellular localization	CNN, RNN BLSTM, FFNN, Attention models	Lasagne, Theano, Deep Loc: http://www.cbs.dtu.dk/services/DeepLoc	Python	License MIT	90	Supervised Learning: Classification	Protein structure prediction
Vang Y., 2017, US (Vang and Xie, 2017)	IEDB MHCBN SYFPEITHI	Human leukocyte antigen (HLA) complex	HLA class I-peptide-binding prediction	NLP, CNN	Keras, Theano, https://github.com/uci-cbcl/HLA-bind	Python	Open Source	100	Supervised Learning: Regression	Protein structure prediction
Wang S., 2017, US (Wang et al., 2017)	Pfam CASP CAMEO	150 Pfam families 105 CASP11 test proteins 76 hard CAMEO	5f5pH	DRNN	TensorFlow, Theano http://raptorx.uchicago.edu/ContactMap/	Python	Apache 2.0	75	Supervised Learning: Classification Regression	Protein structure prediction
Yang J., 2015, China, US (Yang et al., 2015)	PDB SPx dataset PDBCYS dataset	Amino acid sequence	Structure prediction of cysteine-rich proteins	HMM, SVR	CYSCON http://www.csbio.sjtu.edu.cn/bioinf/Cyscon/	N/A	N/A	75	Supervised Learning: Regression	Protein structure prediction
Li Z., 2014, US (Li et al., 2014)	PISCES	TL2282 dataset TS500 dataset TR1532 dataset	Sequence profile prediction	SPIN, NN	SPIN http://sparks-lab.org	Python, Linux	Open Source	85	Supervised Learning: Classification	Protein structure prediction
Wong K., 2013, Canada, US, Saudi Arabia (Wong et al., 2013)	Protein-Binding Microarray dataset	DNA sequence	DNA-motif discovery	Kmer-HMM	kmerHMM http://www.cs.toronto.edu/wkc/kmerHMM	N/A	N/A	50	Supervised Learning: Classification. Unsupervised Learning: Clustering	Model Discovery
Katzman S., 2008, US (Katzman et al., 2008)	PDB PISCES	Amino acid sequence of a protein of unknown structure	Local structure prediction	Multi-layer NN	PREDICT-2^ND^ http://www.soe.ucsc.edu/∼karplus/predict-2nd/	C++	Open source	80	Unsupervised Learning: Clustering	Protein structure prediction
Bindslev C., 2002, Denmark (Bindslev-Jensen et al., 2003)	20 Patients with allergy to Macrozoarces americanus	Macrozoarces americanus	Investigate potential allergenicity of Ice Structuring Protein (ISP)	DT	N/A	N/A	N/A	45	Supervised Learning: Regression	Protein structure prediction

3D-CNN, three-dimensional convolutional neural network; ACNN, Asymmetric convolutional neural network; BLSTM, Bidirectional long short-term memory; BRNN, Bidirectional recurrent neural network; CNN, Convolutional neural network; Deep3I, Deep inception-inside-inception network; DNN, Deep neural network; DRNN, Deep residual neural network; DT, Decision Tree; FFNN, Feed forward neural network; HMM, Hidden Markov model; K-merHMM, K.mer Hidden Markov model; LSTM, Long short-term memory; MC, Monte Carlo; ML, Model; MLP, Multilayer perceptron; NN, Neural network; PSO, Particle swarm optimization; RNN, Recurrent neural network; RNN 2, Residual neural network; SPIN, Sequence Profiles by Integrated Neural network; SVR, Support vector regression; UDNN, Ultradeep neural network.

**TABLE 6:** An overview of the protein contact map prediction, protein-binding prediction, protein site prediction, and genomics articles with the quality assessment.

First Author/Year of Publication/Country	Database	Initial scaffold (ID)	Designed Protein	ML model	Software/Sever	Programming language/Platform	License	Quality (%)	Machine learning	Protein application
	Protein Contact Map Prediction									
Yang H., 2020, China (Yang H. et al., 2020)	SCOPe 2.07	N/A	Contact map protein prediction	GAN	Keras, Tensorflow https://github.com/melissaya/GANcon	Python	Open Source	70	Supervised Learning: Regression	Contact map prediction
Hanson J., 2018, Australia, China (Hanson et al., 2018)	PDB UniProt	Primary amino acid sequence, proteins from CASP12	Protein contact map prediction	CNN, 2D-BRLSTM	http://sparks-lab.org/jack/server/SPOTContact/	N/A	N/A	95	Supervised Learning: Prediction	Protein contact map prediction
Ashkenazy H., 2011, Israel (Ashkenazy et al., 2011)	PDB	3D protein structure	Contact map prediction	WMC	http://tau.ac.il/∼haimash/WMC	Perl	Open Source	45	N/A	Protein map prediction
Durrant J., 2011, US (Durrant and McCammon, 2011)	PDB MOAD	Crystal structure data	Identification of small-molecule ligands	ANN scoring function map	NNScore 2.0 http://www.nbcr.net/software/nnscore/	Python	Open Source	50	Supervised Learning: Classification	Protein map prediction
Lin G., 2010, US (Lin et al., 2010)	PDB	Protein Folding Rates. Predicting protein folding rates from geometric contact and amino acid sequence	Protein folding kinetic rate and real-value folding rate	SVM, SVR	SeqRate http://casp.rnet.missouri.edu/fold_rate/index.html	Java	Open Source	75	Supervised Learning: Classification	Protein map prediction
	Protein-Binding Prediction									
Song J., 2021, China (Song et al., 2021)	PDB Swiss-Prot	ATP-binding proteins	Protein–ATP-Binding Residues	DCNN, LightGBM	TensorFlow, Keras https://github.com/tlsjz/ATPensemble	Python	Open Source	80	Supervised Learning: Regression Prediction Classification	Prediction of Protein–ATP Binding Residues
Kwon Y., 2020, Korea (Kwon et al., 2020)	PDBind-2016	VEGFR2 kinase domain and adenosine deaminase	Prediction of affinity-binding of a protein–ligand complex	3D-CNN	Keras, Tensorflow	Python	Open Source	85	Supervised Learning: Prediction	Protein affinity-binding prediction
Mahmoud A., 2020, Switzerland, US (Mahmoud et al., 2020)	PDB	HIV-1 protease, dihydrofolate reductase	Hydration site occupancy and thermodynamics predictions	CNN	https://hub.docker.com/r/lilllab/watsite3	N/A	Open Source	65	Supervised Learning: Regression Classification	Protein–ligand-binding prediction
Wang M., 2020, US (Wang M. et al., 2020a)	SKEMPI 1.0, 2.0 dataset AB-Bind S645 dataset	Protein–protein complexes	Protein–ligand-binding affinity predictions	Site-specific persistent homology, CNN, GBT	TopNetTree, Keras https://doi.org/10.24433/CO.0537487.v1	Matlab, java, python	Open Source	90	Supervised Learning: Prediction	Protein–protein-binding affinity
Luo X., 2019, China (Luo et al., 2020)	Transfac	DNA sequences	predicting DNA–protein binding	CNN	Keras, Tensorflow https://github.com/gao-lab/ePooling	Python	Open Source	70	Supervised Learning: Regression Prediction	Protein-binding prediction
	Protein Site Prediction									
Zheng W., 2020, China, US (Zheng et al., 2020)	SCOPe2.07	N/A	Protein domain boundaries	DRNN	https://zhanglab.ccmb.med.umich.edu/FUpred/	N/A	Open Source	60	Supervised Learning: Classification	Protein domain identification
Cui Y., 2019, China (Cui et al., 2019)	BioLip	Fourteen binding residues	Protein–ligand-binding residue prediction	DCNN	TensorFlow, https://github.com/yfCuiFaith/DeepCSeqSite	Python	Open Source	100	Supervised Learning: Prediction	Protein site prediction
Fu H., 2019, China (Fu et al., 2019)	PLMD	Sequences and physicochemical properties of protein	Predict Lysine ubiquitination sites in large scale	CNN, DL DeepUbi	TensorFlow, DeepUbi: https://github.com/Sunmile/DeepUbi	Python, MATLAB, Linux	Open Source	100	Supervised Learning: Classification	Protein site prediction
Haberal I., 2019,, Norway, Turkey (Haberal and Ogul, 2019)	PDB	Metal binding of histidine and Cysteine amino acids	Prediction of metal binding in proteins	2D-CNN, LSTM, RNN	Keras, TensorFlow	Python	Open Source	100	Supervised Learning: Prediction	Protein site prediction
Savojardo C., 2019, Italy (Savojardo et al., 2020b)	UniprotKB/Swiss-Prot	Mitochondrial proteins	Sub-mitochondrial cellular localization	CNN	http://busca.biocomp.unibo.it/deepmito	Python	Open Source	75	Supervised Learning: Regression	Protein sub-mitochondrial site prediction
Simha R., 2015, Canada, Germany, US (Simha et al., 2015)	DBMLoc dataset	N/A	Protein multi-location prediction	MDLoc, BN	MDLoc http://www.eecis.udel.edu/compbio/mdloc	Python	Open Source	75	Supervised Learning: Classification	Protein site prediction
Briesemeister S., 2010, Germany (Briesemeister et al., 2010)	UniProt	Protein sequence	Predict protein subcellular localization	NB	Yloc Weka www.multiloc.org/YLoc	Python, Java, Linux	Open source	85	Supervised Learning: Classification	Protein site prediction
Huang W., 2008, Taiwan (Huang et al., 2008)	UniProt GO	SCL12, SCL16 Sequence-based, GO terms, protein sequence	Prediction method for predicting subcellular localization of novel proteins	GA, SVM	LIBSVM ProlocGO http://iclab.life.nctu.edu.tw/prolocgo	N/A	N/A	75	Supervised Learning: Classification	Protein site prediction
Ladunga I., 1991, Hungary (Ladunga et al., 1991)	UniProt	Signal peptide	Novel predicted signal peptides	NN (Tiling algorithm)	N/A	C	N/A	50	Supervised Learning: Classification	Protein site prediction
	Genomics									
Dai W., China, 2020 (Dai et al., 2020)	Reactome DB and InBio Map DB	Human essential gene	Predict human essential genes	Network embedding, SVM	N/A	N/A	N/A	50	Supervised Learning: Classification	Human gene prediction
Picart-Armada S., 2019, Belguim, UK, Spain (Picart-Armada et al., 2019)	STRING	Gene-disease data from 22 common non-cancerous diseases	Target disease gene identification	PR, Random Randomraw EGAD, PPR, Raw, GM, MC, Z-scores, KNN, WSLD, COSNet, bagSVM, RF, SVM	https://github.com/b2slab/genedise	R	Open Source	80	Semi-supervised, Supervised Learning: Classification	Target gene identification, target drug discovery

2D-BRLSTM, two-dimensional bidirectional Res-long short-term memory; 2D-CNN, Two-dimensional convolutional neural ubcell; 3D-CNN, Three-dimensional convolutional neural ubcell; ANN, Artificial neural network; BN, Bayesian Network; CNN, Convolutional neural network; DCNN, Deep Convolutional neural network; DL, Deep learning; GAs, Genetic algorithms; GBT, Gradient boost tree; KNN, k-nearest neighbor; LightGBM, Light Gradient Boosting Machine; LSTM, Long short-term memory; NB, Naïve Bayes; NN, Neural network; RNN, Recurrent neural network; SVM, Support vector machine; SVR, Support vector regression; WMC, Weighted multiple conformation.

## Results

### Article Scaffolding

This article is arranged as follows (Figure 2): first, we provide a representation of the process in designing, preparing, and describing of the guideline throughout the article. Secondly, we review the presented formulation of the research question toward the determined problem formulation and objectives of the research, including the treatment of the data and the applications of it. Thirdly, the article processes the observation, research, and review of a series of articles to further study the data obtained and review similarities. Furthermore, the gathering of AI–PS information, within this processing of the identification of filtered data, curated data and features implemented, the observation of input data, data encoding format, recording of machine learning algorithms and methods, as so the post-processing treatment, quality rule processing, filtering, combination, or unification of information, which passes into the interpretation of the information recollected, and representation of it by the usage of figures and tables, portrays the results, which are focused on the latest findings of AI applications in the field of protein science as well as the usage of specific algorithms for protein design. Therefore, this aims to include a wide-scope range of the state of the art of artificial intelligence within protein science; this leads us to a latter analysis and discussion regarding the identification and prediction of AI applications into the protein field, by classification and identification of main protein structures, and other components not found or described yet in nature, and the resolution of possible protein prediction structures and other components of them are plausible outcomes of future research.

### Toward an Innovative Cross-Functional AI–PS Binomial Inter-field

This systematic review and meta-analysis are focused on the latest findings of AI applications to the field of protein science as well as specific algorithms used for protein design. Furthermore, it aims to include a wide scope of the state of the art of artificial intelligence in protein science. PIO is the methodology used to address the following research question: What is the state of the art in the use of artificial intelligence in the protein science field? Figure 1 shows the total number of articles retrieved using the PIO strategy in the PubMed database.

The systematic review process began with 541 references obtained from five electronic databases: 42 were from PubMed, 74 were from Ebsco, 48 were from Bireme, 38 were from OVID, and 339 were from Web of Science. In the first screening, 403 articles were removed: 250 articles with a double reference; 2 not written in Spanish or English; 149 whose topic was irrelevant to the review; and two newspapers, letters, or reviews. This election process left 138 references, and manually we added 6, thus getting a total of 144 articles for the review (Figure 3).

A second screening (eligibility) was performed using the following set of quality criteria:

1. Clear research questions and objectives.
2. Definition of the measured concepts.
3. Reliability and feasibility of the instruments to be measured.
4. Detailed description of the method.
5. Scaffolding and enhanced protein information.
6. Characteristics of scaffolding and its realization.
7. Appropriate system and learning approach.
8. Journal impact.

A total of 93 articles were included for further analysis, and 51 studies were removed based on quality criteria.

### Machine Learning Approach to Protein Science

Proteins are influenced by epigenetic phenomena (cellular stress, aging, *etc*.) because of their multiple structure-folding-function within protein science (PS), phenomena that can be challenged through the use of artificial intelligence (AI).There are several questions within this interdisciplinary approach such as How do proteins evolve? How do proteins fold and get their tridimensional structure? What are their networks within proteins? Given the astronomical numbers of possibilities for protein structures, configurations, and functions that require the use of AI as a tool to fully understand protein behavior.

**FIGURE 5:**
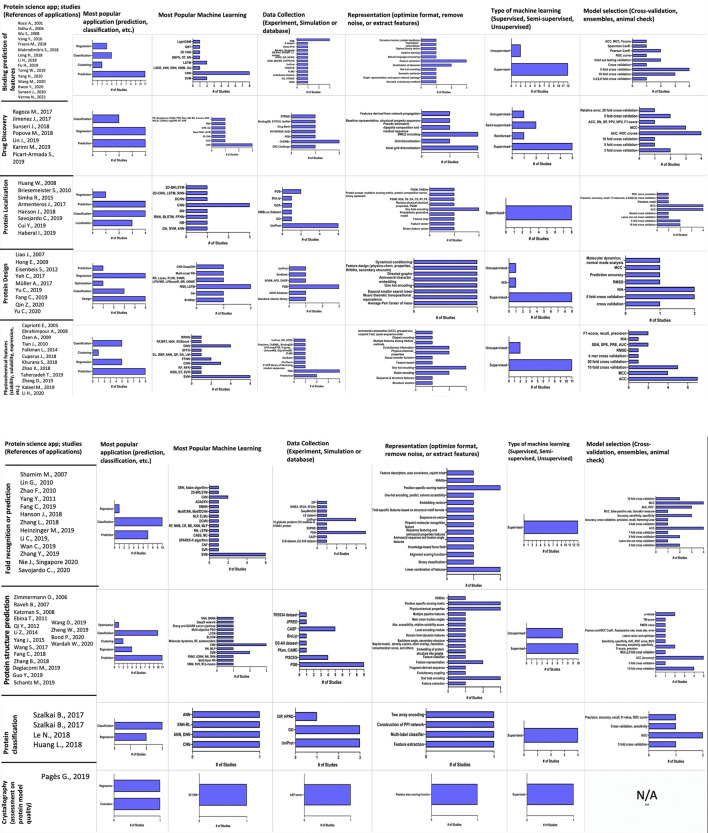
Machine learning and artificial intelligence applications to protein sciences. Information includes the number of studies, applications, databases, methods, and validation used.

**FIGURE 6:**
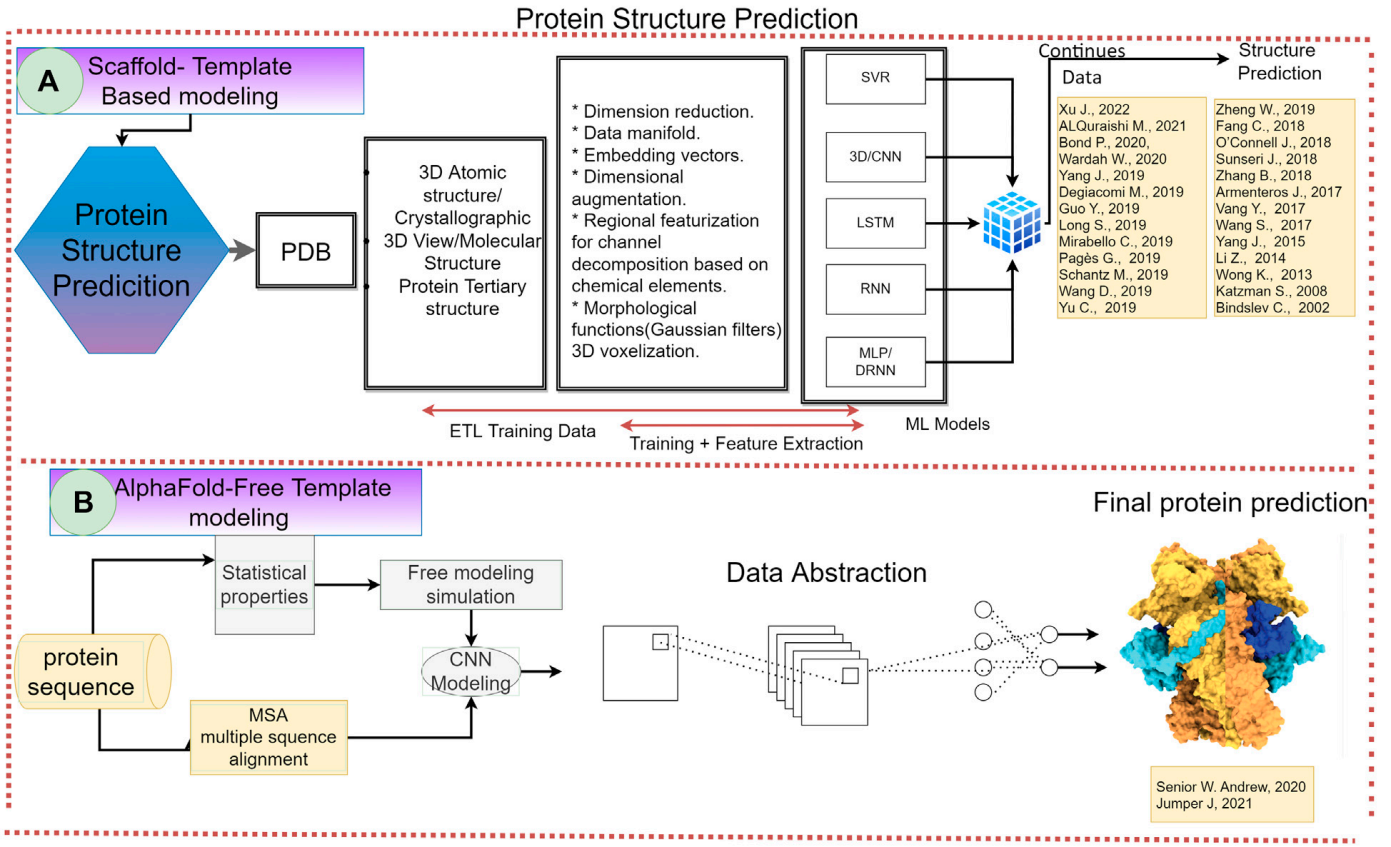
Representation of the specific case for protein structure prediction in the supervised learning framework. Revealing the most common flow followed by the studies analyzed. From extraction, training data, feature extraction procedures and data continuity. Including the PDB database, the most common supervised algorithms, SVM, SVR, 3DCNN.

A total of 144 articles were assessed for quality (Tables 2–6) resulting in 93 articles (Table 1), those articles that were greater or equal to 75 in the quality percentage qualifications were kept for the final biochemical meta-analysis. For this review and meta-analysis, we identified five main applications of AI into PS (Tables 2–6 and Figures 4–6)

I. Protein design and drug design (Table 2)
  a) *De novo* protein design.
  b) Novel biocatalyst design.
  c) Novel function and ligand interaction.
  d) Evolution of non-existent proteins in nature.
  e) Chemical structure and properties.
  f) Drug–drug interaction.
  g) Drug–receptor interaction.
  h) Drug effects.
II. Protein function, function prediction, and novel function (Table 3)
  a) Protein–ligand interactions.
  b) Hydroxylation site prediction.
  c) Prediction of the local properties in proteins.
  d) Enzymatic function prediction.
  e) Predicting protein–protein interactions.
  f) Function prediction.
  g) Molecular property prediction.
III. Fold ID, physicochemical properties, and protein classification (Table 4)
  a) Fold Id.
  b) Glycation site predictor.
  c) Phosphorylation site predictor.
  d) Protein–protein interaction.
  e) Intrinsically disordered protein prediction.
IV. Protein structure prediction (Table 5)
  a) Protein structure prediction: primary, secondary, and 3D-structures; domains, active sites, allosteric sites, and structural feature prediction.
  b) Protein structure classification: folds, structural families, intrinsically disorder proteins, *etc*.
  c) Protein–protein interactions and protein networks.
  d) Protein–ligand interactions: substrates, inhibitors, activators, ions, *etc*.
  V. Protein contact map prediction, protein-binding prediction, protein site prediction, and genomics (Table 6)
  1) Contact map prediction.
  2) Protein sub-mitochondrial site prediction.
  3) Genomics.

The 40% (57/144) of the protein studies by AI applications were the following ones: myoglobin, silk protein, amyloid proteins, Rab family, cathepsin S family, kinases family, K proteinase, barnase, apolipoprotein family, protein DND_4HB, and antimicrobial peptides. Studies in enzymes should be pointed out, oxidoreductases, transferases, hydrolases, lyases, isomerases, ligases, NOS (nitric oxide synthase), lysozyme, which are included in the columns of the initial scaffold (Tables 2–6). These proteins are very useful in the industry as well as in the biomedical fields. With respect to the type of organisms, the more explored are the following ones: *E. coli, Drosophila, Caenorhabditis elegans, Homo sapiens, S. cerevisiae yeast, Mus musculus (mouse), Geobacillus, and Coronavirus.*

Tables 2–6 present the lists of the most commonly used databases in AI applications on PS. Of all the studies reviewed, the single use of main databases and datasets used is as follows:
1) PDB (30/144) 21%.
2) Author’s dataset construction (21/144)15%.
3) UniProt either UniProtKB or UniProtKB/SwissProt (12/144)8%.
4) CASP (critical assessment of protein structure prediction) database (5/144)3%.
5) SCOP (structural classification of proteins) (4/144)3%.
6) N/A, GenBank (4/144) 3%.
7) Protherm (3/144) 2%.
8) BioLip (biologically relevant ligand–protein) (2/144) 1%.
9) PLMD (protein lysine modifications database) (2/144) 1%.
10) And each of the next databases ChEMBL, eSol, GEO, DSSP, Drugbank, BioCreative, Transfac, STRING, BRENDA, SPINE, PISCES, NCBI, D3R Grand challenge 3, and KEGG with a (1/144)1%.

From the studies reviewed, (23/144), 16% use two databases. Of these, the latter (11/23) 48% uses a combination of the PDB and HSPP, PISCES, ProTherm, MOAD, SPx dataset, ChEMBL, DisProt, and UniProt/SwissProt; (4/23)17% use a combination of the GO database with UniProt or STRING; (4/23)17% uses a combination of the UniProt/SwissProt database with ENZYME, DIP, TrEMBL, and CAFA database; and a (2/23)9% combination among DIP, HPRD, SKEMPI database, and SPx dataset. The rest (24/144)17% belongs to a combination of three or more databases with PDB, UniProt, among others.

Moreover, several authors (Shamim et al., 2007; Simha et al., 2015; Yang et al., 2015; Li et al., 2018; Torng and Altman, 2019) focused on using previously constructed datasets, while others chose the creation of their own, based on their own design and outcome, for example, NOS, PPI’s, SPX, DBMLoc, D-B, and Extended D-B (Tables 2–6 and Figure 5).

The following tables show the principal protein categories that were found in this study. Table 2 shows the result of each of the 38 articles that were considered in the protein and drug design category.

Table 3 shows 26 studies that are related to protein function prediction and 6 studies related to function prediction and novel function.

Table 4 shows 19 studies that are related to fold ID and physicochemical properties and 8 studies related to protein classification.

Table 5 shows 26 studies that are related to protein structure prediction.

Table 6 shows five studies for protein contact map prediction, five studies for protein-binding prediction, nine studies for protein site prediction, and two studies for genomics.

Table 1 shows the overview of the extracted information of the selected studies based on the quality criteria.

### Machine Learning Paradigms and AI Algorithm Roles

The most applied approach we found as a result of our review and meta-analysis corresponds to supervised learning (123/144)85%, which focuses on classification algorithms (CNN, NB, KNN, RF, SVM, *etc*.) and regression algorithms (SVR, RFR, DT, ANN, DNN, *etc*.) that are used for a variety of tasks: detection of functional sites, hydroxylation sites, amino acid composition, DNA expression sequences, protein interaction, biomarker finding, protein design, drug design, 3D structure prediction, and protein folding (Tables 2–6 and Figures 4, 5). Within supervised machine learning (123), we found that classification techniques overrule, by far, regression ones (31/123) (for reference, *see* Tables 2–6). On a closer look, we see that these methods are generally very good at prediction tasks, although complexity may be significantly increased by the execution time required, something that is often reported as a drawback of this method (AlQuraishi, 2021).

**FIGURE 7:**
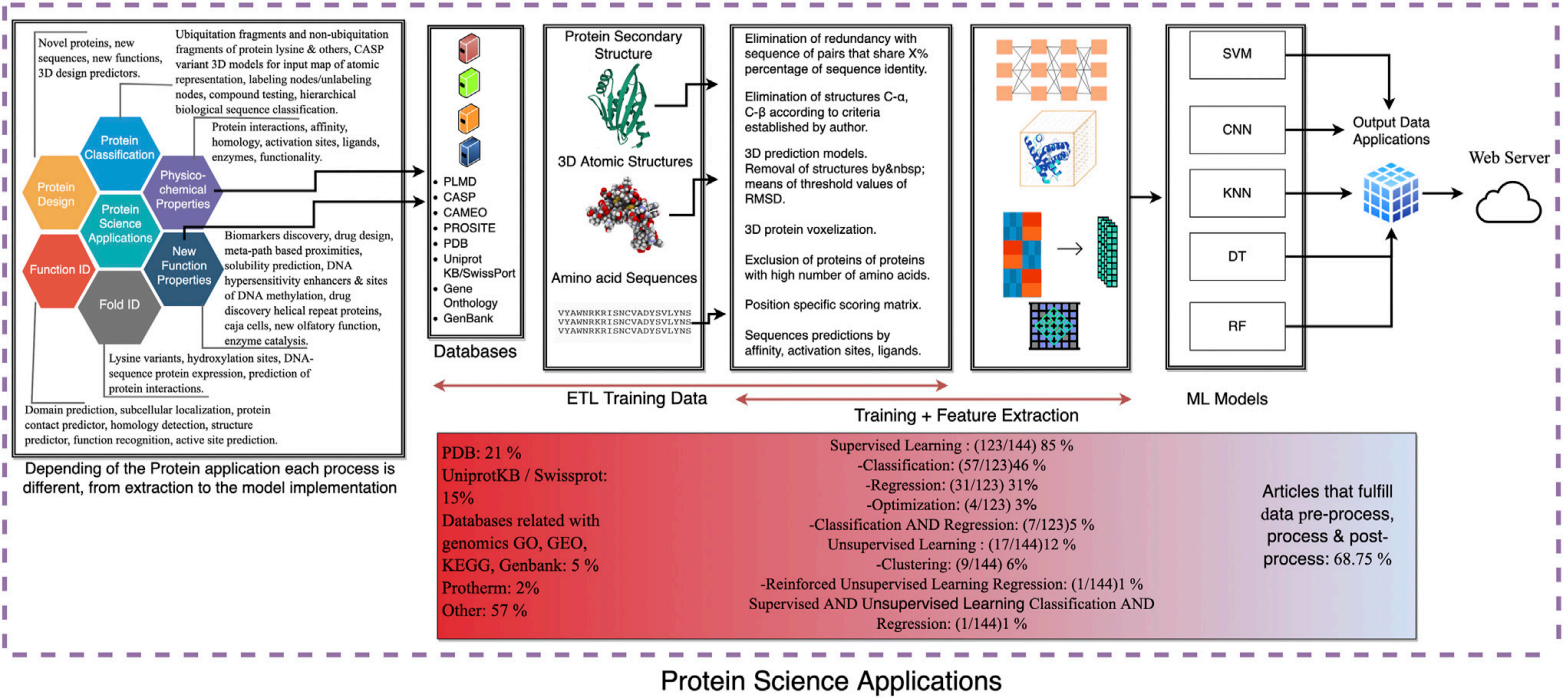
Representation of the whole AI process based on the selected protein application. The process amalgams several steps: protein application (protein design, protein classification, protein prediction, *etc*.), extraction (selection of database), transform (code development and filtering), and load (input of the training data) (ETL) for the training data and the feature extraction procedure is the building of the machine learning network. Outcome step and a proposal server application.

In contrast to supervised learning, it is only (17/144)12% focusing on unsupervised learning, using clustering algorithms (CNN, FFNN, LSDR, DL, HMM, MRF, NN, *etc*.) for various purposes, such as protein solubility prediction, protein prediction of new functions, discovery of DNA motifs, detection of protein structures, and prediction of the nuclear Overhauser effect at low energies. Of the eight articles using this approach, two of them report an improvement in performance as an advantage, one of them in time reduction (Frasca et al., 2018) and the other one in the acceleration of automated protein function prediction methods in general (Makrodimitris et al., 2019). At the same time, however, a disadvantage reported is that time execution may be increased, a fact that should not surprise us, for it is well known that unsupervised learning algorithms are characterized by being computationally very complex methods (Table 1 and Figures 4–7).

On the other hand, supervised machine learning is used just a little more than deep learning techniques. Moreover, it is interesting to note that roughly (77/144)53% of the deep learning articles combine two clustering algorithms: CNN (47/77)61% and LSTM (16/77)21%. Of course, some articles put forward optimization procedures in an algorithmic genetic fashion (Figures 4–7).

Regarding hybrid algorithms using neural networks, we found that all 11 articles explicitly stating their use of hybrid algorithms belong to the deep learning paradigm, combining CNN and LSTM or RNN and CNN. One of them (Almagro Armenteros et al., 2017) goes even further; in that, it uses a combination of these two neural networks to predict protein subcellular localization and then an attention mechanism to identify protein regions important for subcellular localization (Table 1 and Figures 4–6).

It is interesting to note as well that nine articles are used for prediction (glycation product prediction (Chen et al., 2019), protein secondary structure (Guo et al., 2019), prediction of metal binding in proteins (Haberal and Ogul, 2019), compound–protein affinity prediction (Karimi et al., 2019), prediction of protein structural features (Klausen et al., 2019), protein contact map prediction (Hanson et al., 2018), prediction of protein interactions (Huang et al., 2018), predicting hydroxylation sites (Long et al., 2018), and predicting protein subcellular localization (Almagro Armenteros et al., 2017)), of which two perform prediction from original sequences (Almagro Armenteros et al., 2017;Li et al., 2018).

Moreover, one of them highlights that one of its applications is for the design of new drugs and one of them performs this task (Karimi et al., 2019).

It is tempting to put forward the claim that hybrid algorithms in deep learning are very good for prediction tasks as well as for applications in the new drug design. It is noteworthy to mention that these articles belong to the last 3 years of our revision, something that suggests that there is a tendency for the use of hybrid methods in the near future (Table 1).

### AI Training, Validation, and Performance

Validation process allows obtaining a quantitative measure of the models’ efficiency. In this systematic review, several methodologies were used to train and validate in the machine and deep learning proposed by means of hold-out and k-fold cross-validation; The most utilized was the k-fold cross-validation, each one with a different folding proposal, e.g., 2-, 3-, 5-, and 10-fold (Szalkai and Grolmusz, 2018a), trained and validated its algorithm utilizing two validations: 3- and 5-fold cross-validations. Several articles used a graphics processing unit (GPU) that was employed to accelerate the deep learning training and validation process. The most utilized AI algorithm in these articles was CNN, with a 33% occurrence, followed by DNN with 9%, both programmed with Python. The performance of the AI algorithms for protein design was evaluated using parameters such as sensitivity, specificity, true-positive rate, false-positive rate, accuracy, recall, precision, F1-score, area under the curve (AUC), receiver operating characteristic (ROC) curve, and Matthew’s correlation coefficient (MCC). For the case of the hold-out validation, a percentage of the data that is taken and that percentage is randomly removed from the dataset is selected. This methodology, in particular, is computationally very simple; however, it suffers from a high variance because it is not known that data will end up in the test set or in the training one and of the importance that these data might have. In hold-out validation, datasets, which for this review are the databases of proteins, genes, peptides, *etc*. (*see* Tables 2–6 and Figures 4–6)**,** are randomly divided into two partitions with different proportions (50, 70, or 75% training—50, 30, or 25% validation), which are mutually exclusive. The first part of the database is used to feed the input vectors of the methods and train the machine or deep learning algorithms, while the rest is used to evaluate and validate the results obtained with their proposed algorithms. In contrast, with this type of validation technique, hold-out takes a long time for computational processing, especially for large datasets, in particular case, the large protein databases. As a result of our meta-analysis, we found the use of the hold-out methodology to train and validate their AI proposals, as CNN, RNN, LSTM, and FFNN (Tables 1–6 and Figures 4–6) in the prediction of expressions, interactions, and subcellular localization of proteins and also in the prediction of the peptide binding.

Another technique for evaluating the performance of AI methods, particularly for large databases such as protein design, is cross-validation. Cross-validation is a technique used to (generally) obtain the ability of a model to fit an unknown dataset given a collected dataset. In this context, the k-fold cross-validation is an iterative process that consists of dividing the dataset randomly into k groups of approximately the same size. In this sense, although not all possible combinations of sets are examined, an estimate of the average accuracy more than acceptable can be obtained by training the model only k-fold. The first set is used to train the AI models and the other is used to test and validate them, doing this process k times using a different group for validation in the iteration. Although cross-validation is computationally an intensive method of training and validation, its advantages are the reduction of computational time because the process is repeated k times, where all the data are tested once and used for training, maintaining a reduced variance and bias. Of the total 93 articles in this review, 41 of them (47%) used the following cross-validation schemes: leave-one-out, 2-fold, 3-fold, 4-fold, 5-fold, 6-fold, 7-fold, 8-fold, 10-fold, and 20-fold cross-validations. For most of them, the use of 5-fold and 10-fold cross-validations to analyze the performance of their AI proposals predominated, with 16 and 17 articles, respectively. This method was preferred for the evaluation to the performance of CNN and SVM algorithms, with databases such as PBD, ProTherm, UniProt, GO, and ChEMBL. Additionally, in seven articles (17%), they carried out various types of cross-validations to obtain more information on the performance of their proposals. Another variant to evaluate performance was observed in three articles (7%), which combined the use of both hold-out and cross-validation methodologies in their proposals, which provide them more effective comparison of results in terms of validation schemes.

In contrast, in 22 articles of this review, 25% did not mention neither their training methods nor the validation performed to evaluate the performance of their algorithms used. Likewise, 7% of the articles evaluated their methods using various types of cross-validations at the same time to obtain more information on the performance of their proposals, e.g., 4-fold, 6-fold, 8-fold, and 1-fold, or 3-fold, 5-fold, 7-fold, and 1-fold, or 10- and 20-fold, for databases of PDB, UniProt, GO, ChEMBL, ProTherm, PISCES, GenBank, STRING, and new databases as NOS, SPx, D-B, and Ext D-B.

In general, the performance of all proposed AI algorithms was evaluated using several parameters such as sensitivity, specificity, true-positive rate, false-positive rate, accuracy, recall, precision, root-mean-square error (RMSE), *R* ^2^, F1-score, area under the curve (AUC), receiver operating characteristic (ROC) curve, and Matthew’s correlation coefficient (MCC) (Table 1).

Of the 87 articles selected as finalists, we have the following: 32 use one single algorithm and 55 use a combination of two or three algorithms sequentially. In machine learning, we found 30; in deep learning, we found 20 applying machine learning (SVM); 11 deep learning (RNN); and 6 using optimization through genetic algorithms.

Regarding the programming language in which each study was developed, we found 47 articles do not specify what language they are based on, 75 articles are based on the Python language, of which 57 are based entirely on Python and 18 are in combination with other software; *see* Tables 2–6.

Twelve articles are based on the C++ language of which only three are based exclusively on that language and nine in combination with Python, with C, R, and CUDA and C++ language in the Linux environment.

Other nine articles are based on MATLAB of which only four are based exclusively on that language and five in combination in conjunction with Python and Bioinformatics and with Python and C++.

Six articles are based on the C language of which three are based exclusively on that language and three in combination in conjunction with C++, R, and CUDA, with Java and Python and one with Linux and Windows environment.

Finally, seven articles are based on the Java language of which two are written exclusively in this language and five in combination with TensorFlow and with C and Python.

Regarding software licenses, 90 articles were found to be Open Source. An article is licensed by Neural Power version 2.5. One article specifies an open license type belonging to IBM and GNU, respectively. Unfortunately, 45 items did not specify the type of license they own.

### Road Map of Artificial Intelligence in Protein Science

The goal of this analysis is to provide a road map to apply machine learning and AI techniques in protein science. One of the results of our meta-analysis, for example, in protein structure prediction, is shown in Figure 6 in which we can observe the two main strategies for protein structure prediction. In Figure 2, we show the scaffold-template-based modeling that is the most commonly used for the scientist in this field with very good results. However, recently Senior and collaborators using a free modeling approach successfully developed an *AlphaFold* algorithm using a deep neural network. They generated an outstanding accuracy of the 3D structure of a protein with an unknown fold in CASP14 (Senior et al., 2020). This led to an unsolved big question about the importance of the starting point in protein structure prediction, in particular, and in protein science, in general.

The road map of this research is an evolving and a dynamic process (Figure 7). It begins by obtaining information from a list of several databases, followed by a pre-treatment step over the extracted data, including those steps for eliminating redundancies within sequences, structure threshold based on RMSD values, and the like. Further steps contribute to the required pre-processing to complete the reporting process, and then proceed to the data process of the information itself, which includes the input data and the application of the machine learning algorithm, in which the input data are set to be processed into FASTA sequences, training sets, or 3D structures, depending on the function of algorithm in turn. The algorithms used fall into four categories: supervised learning, unsupervised learning, deep learning, and optimization, where each of these categories include a set of their own subparts, which are then combined and configured to predict new ways to model previous data and contribute to future implementations in protein science. The post-processing of data and the support of the new data acquired are made up of models and sequences that were loaded on the platforms to servers such as “DeepUbi, DeepSol, COSNet, Gnina, among others”, in which these servers are used for the storage or implementation of their respective methods. Figure 7 shows that more than half of the reported research completed the three pre-process, process and post-process steps we set forward, so this sequence may be applied to protein science including protein design, classification, physicochemical properties, functionalities, folding properties, and new functions such as homology prediction, domain prediction, subcellular localization, drug design, sensitivity, and other enhancers that can provide new catalysts and new functions, all of which provide any future development for biomolecular enhancement within protein science through machine learning. Model development is intrinsically related to the protein application to be developed. Data extraction varies depending on the architecture of the model to be developed since the data become more complex as the transformation, training, and feature extraction process unfold. The extraction ranges from obtaining the amino acid sequence, secondary structure to the 3D atomic model, using the atomic coordinates. Transforming data emphasizes on performing an adequate filtering for the use of the information for the training of the model, which leads to the feature extraction for the use of machine learning model and finally generating a final output. The process road map includes the fusion of these different applied AI learnings, models, and classifications into a connected deep learning layer that will be included in future research and test datasets to cover the terms of AI science, proteins, and their applications.

## Final Discussion and Further Challenges for Our Understanding of Protein Science Using AI

### Novelties and Future Direction in the Binomial PS-IA Research

The protein science field has great expectations on ML methods as indispensable tools for the biomedical sciences as well as for the chemical and biotechnology industry, for applied research is moving toward synthetic organisms with artificial metabolic networks, regulators, and so on, creating synthetic molecular factories. The binomial PS-IA research is evolving and strengthening, as shown in the Results section (Tables 1–6 and Figures 4–7). Our research reveals that road maps are most needed to solve complex problems in PS, guiding the exploration into the protein universe. As depicted in Table 1, ML techniques, which are used nowadays, are tailored to the expected results; Tables 1–6 display an array of networks of several solving problem methods, hence showing that guidance is needed in the form of road maps.

It is important to emphasize that in order to design a model algorithm bank functioning as a kit-tool, it is essential to understand the source from which the data are obtained and then used to train each model. The studies analyzed solve classification, regression, and optimization problems. As depicted in Table 1, models providing a solution make use of probabilistic inference, functions, activation functions, reduction of the hierarchical order, and logical inference. These results support the fact that machine learning models are heterogeneous, time demanding to design, and correctly evaluate complex models—since the result may not always be as expected or the method may not be carried out successfully. As illustrated in Table 3, there are some physical limitations blocking the full execution of the various models or algorithms, for example, when there is no appropriate computational equipment. Not surprisingly, several authors report that executing a model requires a high demand on execution time, computational power, extensive time to correctly evaluate the model, large memory consumption, and optimization toward GPUs (Frasca et al., 2018; Almagro Armenteros et al., 2017; Yeh et al., 2018; Jiménez et al., 2017; Lin et al., 2010). Another crucial aspect mentioned in Table 1 is the lack of input data to train the model, something that influences the model’s precision and accuracy (Pagès et al., 2019; Cuperus et al., 2017; Folkman et al., 2014; Qi et al., 2012). Moreover, there are also limitations in model construction, such as errors in the training process, manual intervention of data, overadjustment of the model, and an inadequate algorithm construction. In the studies analyzed, there are cases in which there is no description regarding the performance of the comprehensive models, generating gaps in the understanding of the behavior of the algorithms or models, like whether they are deterministic (Long et al., 2018; Ragoza et al., 2017; Makrodimitris et al., 2019). As stated in the ML and AI Algorithm section, supervised learning is the most used method, something that highlights the use of classification algorithms. Moreover, there seems to be a current trend to solve problems in protein science using techniques that require a cross-functional group of scientists, something that, in turn, highlights the fact that there is plenty of unexplored terrain in the use of unsupervised machine learning.

An interesting finding is the implementation of free code and software, as shown in the AI Training, Validation, and Performance section. Our results exhibit a tendency to create models with transparency, which means that every study implemented in a public server has access to all new models created. Another crucial result is the one depicted in the Road Map of Artificial Intelligence in Protein Science section, which is an abstraction that reduces the design of an artificial intelligence model to be used in the resolution of a specific problem in protein science. The whole process follows three steps directed to build a competent model; these steps are *1*) the procedure to obtain raw data and which type of processing should be followed for the model to be adequate, *2*) the type of algorithm that may be used depending on the complexity of the problem, and finally, *3*) the interpretation of results.

Overall, AI displays a window of opportunities to solve complex problems in PS because of its potential in finding patterns and correlating information that requires the integration of protein data exceeding many petabytes. However, we are still far away from solving all the protein tasks computationally. As a result of our biochemical meta-analysis, we showed that AI applications are strongly directed to function identification and protein classification (Tables 1–6), for machine learning models and methods are heterogeneous and do not always draw a clear line as to whether a process should go in a certain sequence (Table 1 and Figures 4–7). It should also be noted that there is no optimal method, which is why applications have different purposes and conditions, suggesting that algorithms must be customized based on the expected outcome or query (Table 1).

The evaluation accuracy horizon is an open epistemic horizon, as shown in Table 1: the metrics for ML methods used in several applications are limited; there are no reported research articles using random forest, in which the cross-validation is unnecessary. In summary, none of the studies reported explicitly use robustly validated methods.

We end by commenting on a key problem in the binomial AI–PS. As well known, it is not possible to work directly with the protein sequences. To tackle this challenge, several studies address this limitation by representing the sequence of a protein as an input to the deep learning model (Almagro Armenteros et al., 2017; Long et al., 2018; Fu et al., 2019). Moreover, given some featured procedures comprising what may be called the *coding architecture*, which is based on creating a specific-weight matrix or a bit vector that represents the sample. This practice was observed in some articles (Cuperus et al., 2017; Jiménez et al., 2017; Khurana et al., 2018; Le et al., 2018) that work with 2D convolutional neural networks in which the authors reported an increase in sensitivity and precision when using indexed datasets. A similar abstraction was observed in 3D convolutional neural networks since the structural representation of a protein is not a rotational invariant; several authors (Jiménez et al., 2017; Ragoza et al., 2017; Hochuli et al., 2018; Pagès et al., 2019; Sunseri et al., 2019; Torng and Altman, 2019) propose using a volumetric map divided into voxels centered on the backbone atoms, representing the physicochemical properties of proteins.

Regarding other review articles along the lines we have followed, the closest we found is the one by Dara et al. (2021). This review article is restricted to drug discovery, one of the five applications we analyzed (genomics, protein structure and function, protein design and evolution, and drug design).

Of a total of 38 articles we presented in Table 2 concerning protein and drug design, only 11 of them were about protein design, so the comparison is not at all fair between these two articles, as far as the analysis of the bibliography analyzed is concerned. However, we share with these authors part of the challenges for researchers in this area: data quality as well as the heterogeneity of databases to be searched for.

Optimization and the characteristics of a prediction must be carried out with a few design considerations, including how to represent the protein data and what type of learning algorithm to use. These form the establishment of a priority acquisition, standard acquisition, *etc*., and the generation of a protein based on a base model, with the aim that one day it would be possible to have controllable predictive models that can read and generate outputs in a consensual terminology, as revised in Hie and Yang (2022). Clearly showing a replacement of conventional methods to the use of machine learning algorithms (neural networks), attributed to improvements in design, computational power, *etc*., the result of a machine learning algorithm is not deterministic, but rather, it is intended to perform transformation functions in relation to the complexity of the data, as depicted in AlQuraishi (2021). There are volumes and volumes of empirical protein data. It is extremely difficult to synthesize such data for correct use in existing algorithms; however, machine learning has helped to compile a large number of methodologies, considering specific assumptions. Nevertheless, most of the empirical methodologies to demonstrate that drugs are safe and effectively continue to be used since there is a gap in the understanding of how the learning transmission of the data to the model is carried out (Dara et al., 2021).

In order to close our reflection as a research team, we believe that a landmark for the epistemic horizon in research is the reassurance that cross-functional groups of scientists from several academic disciplines, in this case including the participation of experts from the natural sciences (organic chemistry, physics and chemistry of proteins, molecular and structural biology, protein engineering, systems biology, microfluid chip engineering, and nanobiotechnology), together with those in computer science (artificial intelligence, knowledge engineering) promote the innovation process in tecno-sciences by combining tacit and explicit knowledge, sharing skills, methodologies, tools, ideas, concepts, experiences, and challenges to fully explore the binomial AI–PS promising area of research (Hey et al., 2019; Mataeimoghadam et al., 2020; Senior et al., 2020; Tsuchiya and Tomii, 2020). A very recent successful case study that highlights this approach is the team of creators of system *Alphafold* (Senior et al., 2020; AlQuraishi, 2021), one which in the CASP (Critical Assessment of Protein Structure Prediction) competition of three-dimensional protein structure modeling were able to determine the 3D structure of a protein from its amino acid sequence. By doing so, this group of researchers solved one of natural science’s open (until now) and most challenging problems using a deep learning approach combining template-based modeling (TBM) and free modeling (FM). The key point is that the neural network prediction encompasses backbone torsion angles and pairwise distances between residues (Senior et al., 2020). At the dawn of the year 2021, this peak of the iceberg brings fresh air and a great power to the protein science field, in particular, and to the life-sciences more broadly, encouraging the new generation of scientists to work as cross-functional teams in order to tackle novel tasks toward the understanding of nature.

One challenge for the binomial AI–PS research area is to tackle the representation of tacit knowledge and include it in the ML algorithms. The relevance of tacit knowledge in the building up of protein science knowledge has come a long way since Polanyi first noted it, extending to different fields in the search for an improvement of their practical skills. In AI, the predominant way of knowledge acquisition and performance is a formal one in which the machine learns and expresses explicitly through guidelines and that works in a focalized mean; the new task alludes to a tacit dimension (Polanyi, 1962), which remains in the edge of attention and incorporates aspects that are taught and learned mostly through practice and in a comprehensive manner (it is context-specific, spreads in the laboratory environment, and comes into play in decision-making.

### Some Conclusions

To sum up, the systematic review and the biochemical meta-analysis offered in this article focused on the enormous innovation that has been made in the binomial AI–PS research, both in its applications and its road maps to solve protein structures and function prediction, protein and drug design, among other tasks. The contribution of this study is 3-fold: firstly, the setup of a cross-functional group in which computer scientists, professionals in biomedicine, and a philosopher constructed a common language and together identified relevant literature in the inter-field of AI–PS and constructed a bridge between the two fields, which can serve as a framework for further research in either area.

Secondly, we stressed the importance of a finer-grain understanding of training and validation methods of ML models and their outcomes, combining databases from several areas of knowledge (life-science experiments, *in silico* simulations, ML, direct evolution approach, *etc*.) that allowed us to classify, stratify, and contribute to the evolving protein science field. Thirdly, we showed that the binomial AI–PS, a *progressive research program*, as Lakatos would say and has still several challenges to tackle, such as the development of a comprehensive machine learning benchmarking enterprise, the experimental confirmation of the structure of the 3D modeling in laboratories, the classification, *etc*., controls the vulnerability of the neural networks, the development of a tool-kit to design novel biocatalysts not found in nature using reverse engineering, human-made metabolic routes, the design of new antibody molecular factory, novel proteostasis systems, the understanding of protein folding and protein-aggregation mechanisms, *etc*. Finally, we suggested that there may be a paradigm shift in the AI–PS research as a result of the recent great outcome of *Alphafold*, encouraging its use to the new generation of scientists.

In any case, what is clear is that a cross-functional group of scientists from several knowledge domains is required to work in coordination for sharing ideas, methodologies, and challenges toward the development of road maps and computational tools, paradigms, tacit, and explicit knowledge to fully explore and close the gap of the binomial AI–PS, a promising research area.

## Data Availability

The raw data supporting the conclusions of this article will be made available by the authors without undue reservation.

## Glossary

1D-CNN: one-dimensional convolutional neural network
2D-BRLSTM: two-dimensional bidirectional recurrent long short-term memory
2D-CNN: Two-dimensional convolutional neural network
3D-CNN: Three-dimensional convolutional neural network
ACNN: Asymmetric convolutional neural network
ADASYN: Adaptive synthetic sampling
AGCT: Alignment genetic causal tree
ANN: Artificial neural network
BBFNN: Biobasis function neural network
BBP: Back back propagation
BLSTM: Bidirectional long short-term memory
BN: Bayesian network
BRNN: Bidirectional recurrent neural network
BroMap: Branch and bound map estimation
BRT: Booster regression tree
CABS: C-alpha–beta side
CFN: Cost function network
CNF: Conditional neural field
CNN: Convolutional neural network
COSNet: Cost-sensitive neural network
DCNN: Deep convolutional neural network
DeepDIN: Deep dense inception network
Deep3I: Deep inception-inside-inception network
DFS: Depth first search
DL: Deep learning
DMNN: Deep mahout neural network
DNN: Deep neural network
DRNN: Deep residual neural network
DROP: Domain linker prediction using optimal feature
DT: Decision tree
DTNN: Deep tensor neural network
EASE-MM: Evolutionary amino acid and structural encodings with multiple models
ELMO: Embeddings from language models
ENN-RL: Evolution neural network-based regularized Laplacian kernel
FFNN: Feed forward neural network
FIBHASH: Fibonacci numbers and hashing table
GA: Genetic algorithms
GAN: Generative adversarial network
GBT: Gradient boost tree
GBDT: Gradient boosted decision tree
GCN: Graph convolutional network
GR: Genetic recombination
HDL: Hybrid deep learning
HMM: Hidden Markov model
HNN: Hopfield neural network
IBP: Incremental back propagation
KeSCANN: Knowledge-enriched self-attention convolutional neural network
K-merHMM: K.mer Hidden Markov model
KNN: k-nearest neighbor
Lasso: Least absolute shrinkage and selection operator
LightGBM: Light gradient boosting machine
LM: Levenberg–Marquardt
LPBoostR: Linear programming boosting regression
LPSVMR: Linear programming support vector machine regression
LR: Logistic regression
LSDR: Label-space dimensionality reduction
LSTM: Long short-term memory
MC: Monte Carlo
ME: Max entropy
ML: Model
MLP: Multilayer perceptron
MNB: Multinomial naïve bayes
MNNN: Multi-scale neighborhood-based neural network
MNPP: Message passing neural network
MotifCNN: Motif convolutional neural network
Motif DNN: Motif deep neural network
MR: Matching loss regression
MRF: Markov random forest
Multimodal DNN: Multimodal deep neural network
NB: Naïve Bayes
NLP: Natural language processing
ORMR: One-norm regularization matching-loss regression
ParCOSNet: Parallel COSNet
PLSR: Partial least-squares regression
PNN: Probabilistic neural network
PS: Protein science
PSO: Particle swarm optimization
PSP: Predict signal pathway
QP: quick prob
ReLeaSE: Reinforcement learning for structural evolution
RF: Random forest
RN: Relational network
RNN: Recurrent neural network
RNN 2: Residual neural network
RR: Ridge regression
SDHINE: Meta path-based heterogeneous information embedding approach
SFFS: Sequential forward floating selection
SGD: Stochastic gradient descent
SPARK-X: Probabilistic-based matching
SPIN: Sequence profiles by integrated neural network
SVM: Support vector machine
SVMR: Support vector machine regression
SVR: Support vector regression
UDNN: Ultradeep neural network
VSA: Virtual screening algorithms
WMC: Weighted multiple conformations
